# Proteomic analysis of endoplasmic reticulum stress responses in rice seeds

**DOI:** 10.1038/srep14255

**Published:** 2015-09-23

**Authors:** Dandan Qian, Lihong Tian, Leqing Qu

**Affiliations:** 1 Key Laboratory of Plant Molecular Physiology, Institute of Botany, the Chinese Academy of Sciences, Beijing 100093, China

## Abstract

The defects in storage proteins secretion in the endosperm of transgenic rice seeds often leads to endoplasmic reticulum (ER) stress, which produces floury and shrunken seeds, but the mechanism of this response remains unclear. We used an iTRAQ-based proteomics analysis of ER-stressed rice seeds due to the endosperm-specific suppression of OsSar1 to identify changes in the protein levels in response to ER stress. ER stress changed the expression of 405 proteins in rice seed by >2.0- fold compared with the wild-type control. Of these proteins, 140 were upregulated and 265 were downregulated. The upregulated proteins were mainly involved in protein modification, transport and degradation, and the downregulated proteins were mainly involved in metabolism and stress/defense responses. A KOBAS analysis revealed that protein-processing in the ER and degradation-related proteasome were the predominant upregulated pathways in the rice endosperm in response to ER stress. Trans-Golgi protein transport was also involved in the ER stress response. Combined with bioinformatic and molecular biology analyses, our proteomic data will facilitate our understanding of the systemic responses to ER stress in rice seeds.

Secretory and transmembrane proteins are synthesized in the endoplasmic reticulum (ER), and then fold and exit the ER. The ER includes a sophisticated protein quality control system (ERQC)^1^,^2^ that detects misfolded proteins and targets them for degradation, a process called ER-associated degradation (ERAD). Under adverse environmental conditions, the expression of misfolded proteins increases and exceeds the capacity for folding and ERAD, which results in ER stress.

ER stress activates the unfolded protein response (UPR) with two arms of the signaling pathway in plants, one that involves the splicing of an mRNA (bZIP60) by a dual protein kinase (IRE1) and another that involves membrane-associated bZIP transcription factors (bZIP17 and bZIP28). ER membrane-localized IRE1 is the most conserved ER stress sensor in eukaryotes. ER stress allows IRE1 to autophosphorylate its kinase domain and thereby activate the ribonuclease domain. The activated IRE1 splices bZIP60 mRNA in an unconventional cytoplasmic manner. The truncated bZIP60 and activated bZIP17 and bZIP28 are released, whereby they relocate to the nucleus and upregulate the UPR genes. The rice homologs of bZIP60, bZIP17 and bZIP28 are OsbZIP50, OsbZIP39 and OsbZIP60, respectively^3^,^4^,^5^. Recent studies have shown that the plant-specific transcription factors NAC TFs are involved in the plant UPR^6^. A membrane-anchored transcription factor NAC089, which expression level is regulated by bZIP28 and bZIP60, participates to ER stress responses and induces programmed cell death gene expression^7^. The plasma membrane NAC062 and the cytosolic NAC103, whose expression are directly regulated by bZIP60, mediate the UPR in *Arabidopsis*^8^,^9^. The discovery of NAC TFs gives a cue that the plant may have unique features compared to yeast or metazoans during the UPR.

Besides factors acting upstream the UPR sensors in plants, little is known about downstream response genes. The current study shows that most ER stress-induced genes are chaperones or protein-folding catalysts, such as binding protein (BiP), protein disulfide isomerase (PDI) and calnexin (CNX). The severe suppression or significant overexpression of BiP1 in rice endosperm leads to ER stress, which reduces the seed storage protein (SSP) content and alters the intracellular structure of the endosperm cells^10^. PDI acts a catalyst of disulfide bond formation and rearrangement and may play a role in storage protein biogenesis^10^,^11^. The absence of endosperm-specific PDI-like protein (PDIL1-1) enhanced proglutelin accumulation in the ER and induced ER stress^12^. CNX is an integral transmembrane protein that selectively binds to the unfolded glycoproteins and prevents misfolded proteins from transporting to the Golgi apparatus^13^. Furthermore, in metazoans, a membrane-associated protein kinase called protein kinase RNA-like endoplasmic reticulum kinase (PERK), which phosphorylates and inactivates a eukaryotic elongation factor, eIF2a, thereby attenuating translation^14^. But so far plant PERK-like proteins have not been identified.

A variety of abiotic and biotic stresses, including salt or heat stress, plant viral movement protein^15^,^16^, and treatment with ER stress agents, such as tunicamycin, dithiothreitol and cyclopiazonic acid, can induce ER stress. The seed acts an ideal platform for producing recombinant proteins^17^, high production of recombinant proteins often causes ER stress because of heavy loading on the ER lumen, which results in floury and shrunken seeds^18^,^19^. Previously reports showed that recombinant antibody production in *Arabidopsis* seeds triggers an unfolded protein response^20^, and the overexpression or suppression of BiP1 in rice endosperm could cause seed-specific ER stress^21^. Tian *et al.* found that the endosperm-specific knockdown of OsSar1 (secretion-associated, Ras-related protein 1) blocked secretory proteins in the ER, which elicited an ER stress response in rice seeds, resulting an opaque and lethal seeds^22^.

The ER stress response is a complex process that maintains the balance between stress adaptation and growth regulation in plants. Although some ER stress response-related factors have been identified via bioinformatic and molecular biology analyses, how these sensors passed down the stress signal to make a life-or-death decision is largely unknown, especially in plants. Proteomics has emerged as a powerful tool to investigate protein changes. Isobaric tags for relative and absolute quantitation (iTRAQ), which are based on the enzymatic digestion of proteins prior to isobaric labeling, are a superior choice in quantitative proteomics due to their high proteome coverage and labeling efficiency^23^. In this study, we used an iTRAQ-based quantitative proteomic analysis of ER-stressed rice seeds due to the endosperm-specific suppression of OsSar1, to identify protein abundance changes in response to ER stress. In order to address the relationship between the differential proteins and ER stress, functional annotation and pathway analysis of the differential proteins were performed. Furthermore, the functions of some of the newly discovered proteins associated with ER stress have been discussed.

## Results and Discussion

### Production of *OsSar1* RNAi transgenic rice

**Figure 1:**
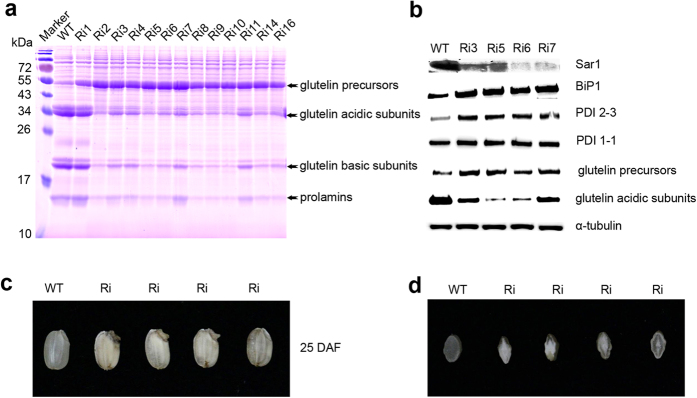
Expression of storage proteins and molecular chaperones in *OsSar1* RNAi transgenic rice seeds. (**A**) SDS-PAGE of storage proteins and (**B**) Western blot analysis of protein levels of Sar1, BiP1, PDI2-3, PDI1-1, glutelin precursors, and glutelin acidic subunits in *OsSar1abc* RNAi-transformed seeds. α-tubulin was a loading control. (**C**) Morphology of *OsSar1abc* RNAi seeds at 25 DAF. (**D**) Transverse sections of grains.

Transgenic rice plants with seed-specific ER stress were produced by suppressing *OsSar1abc* by RNA interference (RNAi) in the endosperm under the control of the *GluA-2* promoter as previously described^22^. We generated 17 independent transformants that were verified with a PCR analysis (data not shown). To investigate the effect of ER stress on the expression of SSP, total protein was extracted from developing seeds 15 days after flowering (DAF) (Fig. 1A) and subjected to SDS-PAGE. Compared with the wild-type control, transgenic seeds showed reduced levels of the glutelin acidic and basic subunits, α-globulin and prolamine, while the levels of the glutelin precursors were increased. A Western blot analysis confirmed the reduced expression of glutelin acidic subunits and the increased expression of the glutelin precursors, OsBiP1 and OsPDIs (Fig. 1B). The transgenic seeds showed an abnormal phenotype, with opaque, floury and shrunken endosperm and lethal embryos, as previously reported (Fig. 1C,D)^22^. Thus, the secretory proteins, including the glutelin precursors and α-globulin, were blocked in the ER in *OsSar1* RNAi seeds, which led to ER stress.

### iTRAQ analysis of low salt-soluble total proteins from WT and *OsSar1abc* RNAi rice

**Figure 2:**
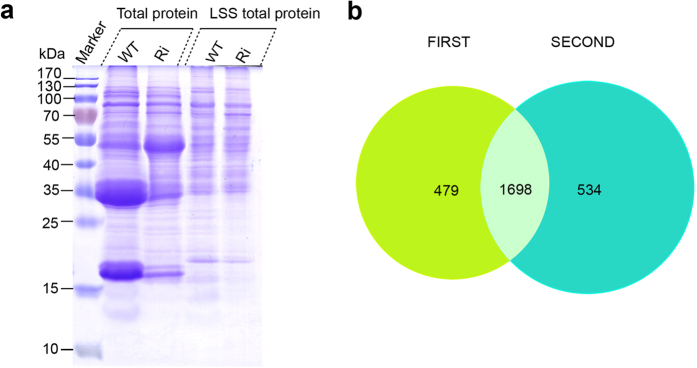
Venn diagram of non-storage proteins identified in rice seed. (**A**) SDS-PAGE of low salt-soluble proteins used for iTRAQ assay. (**B**) Venn diagram of proteins identified in developing seeds from wild-type (WT) and transgenic (Ri) seeds by iTRAQ. Two technical replicates were performed. Green represents the first independent experiment; blue represents technical replicates.

Glutelin, prolamine and globulin are major SSPs that account for approximately 70%, 10% and 5% of the total seed protein in rice, respectively. To eliminate the impact of these proteins, we extracted only low salt-soluble proteins from developing rice seeds^16^. On SDS-PAGE, the low salt-soluble proteins contained fewer SSPs in the WT and *OsSar1* RNAi rice (Fig. 2A), while the levels of other proteins did not markedly differ when compared with the total seed proteins.

We used an iTRAQ analysis of low salt-soluble proteins from developing rice seeds to investigate the ER stress regulation of the proteome. iTRAQ reagents consist of a peptide reactive group, a reporter group, and a balance group. The peptide reactive group specifically reacts with the N-terminus group of a peptide and epsilon amino group of lysines. The reporter group is a tag of varying masses (114, 115, 116, and 117) consists of various combinations of isotopic elements. The balance group ensures that the mass of a labeled peptide remains constant irrespective of the labeling agent, i.e. 114, 115, 116, or 117, to ensure that all labeled peptides are identical in single MS mode. Due to the neutral loss of the balance group, the four diagnostic reporter ions (114, 115, 116, 117) were liberated during LC-MS/MS, and the peak intensities of different reporter ions provided quantitative information on proteins^24^.

In our study, WT samples were labeled with reagents 114 and 116, and transgenic samples were labeled with reagents 115 and 117. These samples were combined into one aliquot. The 116-labeled WT sample and 117-labeled transgenic sample acted as the biological duplicates. The technical duplicates were measured by nanoflow LC-MS/MS. The Protein ID Summaries are given in Supplementary Tables S2 and S4, and the Differential Protein Summaries are given in Supplementary Tables S4-S7. The proteins identified at the critical false discovery rate (FDR) are summarized in Supplementary Tables S8 and S9. In total, 2,711 proteins were identified with an unused ProtScore of ≥4.0. Specifically, 2,177 proteins were identified in the first experiment, and 2,232 were identified in the second experiment, with a total of 1,698 shared identical proteins (Fig. 2B). Finally, 786 differentially expressed proteins were identified from 2 experiments: 574 from the first experiment and 617 from the second experiment. In total, 405 proteins were found to be differentially expressed between the transgenic and WT samples, as determined by a >2.0- fold difference in quantity (p < 0.05). Of these proteins, 140 were upregulated and 265 were downregulated in expression in transgenic seeds (Table 1 and Table S10).

### Protein upregulated in seeds of *OsSar1* RNAi rice

**Figure 3:**
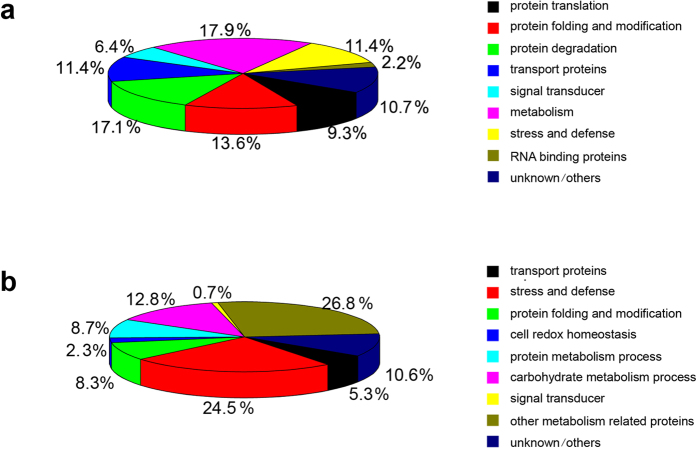
Classification of differentially expressed proteins. (**A**) Functional classes (%) of proteins with >2.0-fold upregulation in *OsSar1* RNAi transgenic rice (p* < *0.05) and (**B**) proteins with >2.0-fold downregulation in *OsSar1* RNAi transgenic rice (p < 0.05).

The 140 upregulated proteins were classified into the following 9 groups using the blast2go software functional annotation and the Rice Genome Annotation Project (http://rice.plantbiology.msu.edu/index.shtml): protein translation (9.3%), protein folding and modification (13.6%), protein degradation (17.1%), transport proteins (11.4%), stress and defense (11.4%), metabolism (17.9%), signal transducer (6.4%), RNA binding protein (2.2%) and unknown/others (10.7%) (Fig. 3A and Table 1). The upregulated ER stress-response proteins were mainly involved in protein translation, protein folding and modification, protein degradation and proteins transport. These results are consistent with previous reports, which stated that ER stress induced the expression of genes involved in protein translation, modification, folding, transport and degradation in *Arabidopsis*^25^. In addition, metabolism-related proteins accounted for a relatively large proportion of the upregulated proteins. The induction of stress-related proteins expression acts as a buffer against stress, and is often associated with metabolic changes. These proteins are also conserved as a fundamentally adaptive cellular response to ER stress in plants. To understand the proteins that respond to ER stress, we described some of the notable differentially upregulated proteins listed in Table 1 in detail.

#### Protein modification and degradation

Protein folding is critical for secretory proteins to exit from the ER, and misfolded proteins are a major contributor to ER stress. To sustain protein-folding homeostasis in the ER, the cell must balance the ER protein folding load with sufficient ER protein folding machinery, particularly chaperones, such as BiP, calnexin, and PDI along with their co-chaperones. The expression of these chaperones increases in response to ER stress to reduce protein misfolding.

BiP, a member of the HSP70 family, is the most abundant chaperone protein in the ER. OsBiP1 is sensitive to ER stress. The overexpression or knockdown of OsBiP1 in rice endosperm can lead to severe ER stress^26^,^27^. OsBiP4 is almost identical to OsBiP5 in amino acid sequence, they cannot be distinguished in iTRAQ experiment. Under normal conditions, OsBiP4 and OsBiP5 (OsBiP4 & 5) are not expressed in any tissue. The OsBiP4 & 5 levels positively correlated with the ER stress levels^28^. In *OsSar1* RNAi seeds, the expression of OsBiP1 (Os02g02410) and OsBiP4 (Os05g35400) increased 5.2- and 28.0- fold, respectively (Table 1). These results stated that the endosperm-specific knockdown of *OsSar1* caused seed-specific ER stress, indicating that those identified differential proteins in present study may be closely associated with ER stress. Besides BiPs, other members of the HSP70 family were also upregulated, such as Os12g32986, Os06g50300, Os07g38760, Os09g07510 and Os02g07120, and the DnaK proteins (Os09g31486, Os02g48110, Os03g16920, Os01g62290, Os03g11910 and Os05g23740) (Table 1 and Table S10), which are cofactors that regulate the binding of BiP to proteins or release BiP from the protein complex^29^. Surprisingly, four of them, Os03g16920, Os01g62290, Os03g11910 and Os05g23740, were downregulated in response to ER stress. Various members of the HSP70 have been reported to be involved in protein import and translocation^30^. Specific interference with Sar1, an important component of COP II complex, blocked the exit of the secretory proteins from ER, these four HSP70 members may play crucial roles in the restablishment of celluar homeostasis by lowering protein load of the stressed ER. These new discovered HSP70 members may have important roles in response to ER stress as well as BiPs.

The rice genome encodes at least 7 OsPDI-like proteins^31^. We found that PDIL1-1 (Os11g09280), PDIL2–3 (Os09g27830), PDIL5–1 (Os03g17860) and PDI (Os03g29240) all showed increased expression in the transgenic seeds (Table 1), while the expression of PDIL1–4 (Os02g01010) decreased approximately 5.5-fold (Table S10). The stress differentially altered the expression of different PDI genes, each PDI may have a distinct function in the ER stress response. The direct oxidation of the active site of PDI1 by Ero1 activity at thiol-disulfide bonds, is essential for native disulfide bond formation within proglutelin^31^. Ero1 (Os03g52340) was upregulated, which indicates that Ero1 coordinates with PDI to promote protein folding during ER stress. Calnexin is an ER chaperone that is involved in the folding of N-glycosylated proteins. Wakasa *et al.* reported that calnexin expression was inversely related to BiP1 expression, because calnexin expression was decreased in ER-stressed rice seeds with OsBiP1 overexpression but increased in OsBiP1 knockdown ER-stressed rice seeds. We found that the expression of both calnexin (Os04g32950) and OsBiP1 (Os02g02410) was increased in ER-stressed seeds with OsSar1 knockdown. In addition, 3 new proteins (Os06g05740, Os01g50030 and Os06g36700) that putatively partake in protein folding and modification have been detected. Os06g36700 belongs to the TCP-1/cpn60 chaperonin-like superfamily, this family includes members from the TCP-1 (T-complex protein) family and the HSP60 chaperone family, which is essential for the correct folding and assemble of subunits into intact complex. The upredulated Os06g36700 in stressed ER suggested that it may act as a novel molecular chaperone to facilitate protein folding during ER stress.

The protein folding machinery attempts to properly fold proteins, however, the capacity of the protein folding machinery is limited. When the folding demands in the ER exceed this capacity, proteins that cannot achieve their native form will accumulate in the ER as aggregates or be eliminated by the ERAD system. Misfolded proteins in the ER are disposed of by the 26S proteasome. In the *OsSar1* transgenic seeds, 6 of the 26S protease-like proteins (Os02g05340, Os08g12820, Os04g36700, Os01g17180, Os03g63430 and Os04g01290) and 7 of the 26S protease regulatory subunits (Os02g10640, Os07g49150, Os02g56000, Os02g21970, Os02g11050, Os02g54340 and Os07g25420) were upregulated (Table 1). These results suggest that the 26S proteasome improved the efficiency of removing the unfolded and unassembled proteins from stressed ER by suppression of OsSar1, which blocked the eixt of the secretory proteins from ER, maintaining cell homeostasis. So far, the knowledge of components and chaperones of 26S proteasome in rice is limited, characterization of the components or chaperones will lead to a better understanding of the ERAD pathway. In addition to 26S proteasome, some protein metabolism-related factors were upregulated in this study, including Os07g31540, Os10g31000, Os07g49520, Os02g55140, Os02g54254, Os12g24080, Os05g41460, Os01g68670, Os05g28280, Os03g31400 and Os09g33780) (Table 1). Os09g33780 showed the most significant increase among them, with a 26-fold change. Os09g33780 belongs to the Fes1 superfamily, has nucleotide exchange factor activity, and the yeast homologue acts as a cytosolic triaging factor that promotes proteasomal degradation of misfolded proteins^32^. This result has an implication that Os09g33780 may be essential for ubiquitin-dependent degradation of misfolded proteins.

Some translation-related proteins were upregulated in *OsSar1* transgenic rice, including 6 tRNA synthetases (Os06g43760, Os05g05840, Os10g10244, Os06g31210, Os05g08990 and Os05g48510), 2 translation initiation factors (Os07g36940 and Os03g18510), 2 elongation factors (Os03g29260 and Os04g02820), 2 ribosomal proteins (Os01g47660 and Os09g31180) and a DEAD-box ATP-dependent RNA helicase (Os06g48750). The primary function of the aminoacyl-tRNA synthetases is to couple tRNAs to corresponding amino acids, participating in protein translation. In mammalian cells, protein kinase RNA-like ER kinase (PERK) phosphorylates and inactivates a translation initiation factor, eIF2a, and thereby slows translation in response to stress^14^. However, this pathway has not been verified in plants. We found that 6 tRNA synthetases and 2 translation initiation factors were upregulated but not downregulated under ER stress, these results are consistent with previous report that the aminoacyl-tRNA synthetases showed increased activity after tunicamycin-induced ER stress^33^,^34^. These factors might enhance the translation of upregulated proteins in response to ER stress. The relationship between protein translation and ER stress responses in plants requires further investigation.

#### Transport proteins and signal transduction

Misfolded proteins in the ER are disposed of in the cytosol by the proteasome or the lytic vacuole^35^, therefore, these proteins must be transported to the cytoplasm or lytic vacuoles for degradation. Sixteen transport related proteins were upregulated in *OsSar1abc* knocked-down endosperm, including a Ras-related protein (Os02g37420), a Sec23/Sec24 trunk domain-containing protein (Os11g24560), a Sec24-like CEF (Os11g29200), 2 clathrin heavy chains (Os11g01380 and Os12g01390), 3 importin subunits (Os05g28510, Os01g13430 and Os05g06350), a ThiF domain-containing protein (Os02g30310) and BRI1-KD interacting protein 103 (Os09g17730). Ras-related protein is a small GTP-binding protein that is involved in the ER-to-Golgi vesicle-mediated transport. Sec23 and Sec24 are components of the COP II complex response during the ER-to-Golgi transport of secretory proteins. Clathrin-coated vesicles are involved in multiple steps during post-Golgi trafficking^36^. ThiF domain-containing protein and BRI1-KD interacting protein 103 in *Arabidopsis* were found to be involved in intracellular protein transport^37^,^38^. ATGRIP/GRIP (Os07g28940) and vacuolar ATP synthase subunit C (Os05g51530) are involved in Golgi vesicle transport^39^,^40^. Notably, the expression of vacuolar-sorting protein Bro1 (Os10g35250) increased 7.8-fold in the ER-stressed seeds, thus, this protein might play an important role in the ER stress response. Outer membrane protein (Os03g16440) plays a role in the targeting of proteins to chloroplasts. Thus, the factors involved in Golgi-derived vesicle transport might contribute to the ER stress responses.

When ER stress is sensed, the membrane-bound IRE1 and OsbZIP39 transducers transduce the stress signals. Like its *Arabidopsis* counterpart, rice IRE1 catalyzes the unconventional splicing of Os*bZIP50* mRNA to activate a transcription factor in response to ER stress^26^,^41^. The active bZIP50 is recruited to the nucleus to initiate the transcription of UPR-associated genes in rice^41^. During ER stress, OsbZIP39 (bZIP17) and OsbZIP60 (bZIP28) are transported from the ER to the Golgi via the COPII complex, where they undergo sequential cleavage by S1P and S2P. The released cytoplasmic components then relocate to the nucleus to activate the expression of ER stress-response genes^42^. However, we could not detect these proteins in our iTRAQ data. We suppose that the translocation of OsbZIP39 and OsbZIP60 from the ER to the Golgi might be disturbed in *Sar1* RNAi transgenic plants, which results in a blockage in the ER and elimination by ERAD^43^. We found that 2 of the 14–3–3-like proteins (Os08g33370 and Os03g50290), a nucleoside diphosphate kinase (Os05g51700) and a BRI1-associated receptor kinase 1 precursor (Os01g59440), were upregulated. These proteins both are components of signaling pathways (Table 1). 14–3–3 proteins are implicated in stress responses in rice^44^. We identified 2 WD domain and G-beta repeat domain-containing proteins (Os07g03160 and Os07g46370), a pleckstrin homology domain-containing protein (Os03g46340) and an AIG1 family protein (Os01g25450). WD domain proteins have been demonstrated having critical roles in signal transduction, several human diseases have been recognized due to mutations in WD-repeat proteins^45^. In addition, sequence alignment showed that WD domain proteins were highly conserved in eukaryotes, indicating that the WD domain proteins in plants may also play vital roles in signal transduction. Despite advances in understanding of the functions of WD domain proteins in human, the functional characterization in rice has been extremely slow. If more such proteins have been identified, a better mechanistic understanding of the signaling pathways in rice will be realized.

#### Proteins related to metabolism, stress and defense

ER stress regulates many proteins that have no obvious direct relationship in protein folding, notable proteins are those involved in essentially all aspects of energy metabolism^46^. Wang and colleagues provided a direct mechanistic link between ER stress and the regulation of carbohydrate metabolism^47^. Microarray analyses also revealed the altered expression patterns of some starch synthesis-related genes in ER-stressed seeds due to the endosperm-specific overexpression or knockdown of BiP in rice^21^. We identified 11 carbohydrate metabolism-related proteins, including Os03g28330, Os08g20660, Os05g09500, Os05g33570, Os05g51670, Os03g09250, Os07g08430, Os07g42960, Os06g30310, Os04g42580 and Os03g61920, which play important roles in sucrose synthesis, the phosphorylation of starch, glycolysis, galactolipid biosynthesis^48^, the glyoxylate pathway and the citric acid (TCA) cycle, respectively. ER acts as an essential “nutrient-sensing” apparatus, playing vital roles in the coordination of metabolic responses via its ability to regulate the synthetic and catabolic pathways of various nutrients. ER is sensitive to energy fluctuations and substrate metabolism, for example, the glucose synthesis or breakdown pathways are transcriptionally regulated during UPR^49^. Interestingly, these proteins above mentioned in our study were reported for the first time in the numerous studies on gene expression changes during ER stress, which might lay the foundation for the future research of the effects of ER stress on carbohydrate metabolism in seeds. Sucrose is required for plant growth and development, Os03g28330 act as a sucrose synthase upregulated in response to ER stress, which may be relevant to the issue that ER stress produces floury and shrunken seeds.

Besides carbohydrate metabolism-related proteins, there are other metabolism-related proteins have also been detected, 3-Dehydroquinate synthase (Os09g36800) catalyzes the conversion of DAHP to DHQ in the shikimate pathway for aromatic compound synthesis. 3-Hydroxyacyl-CoA dehydrogenase (Os01g24680) primarily participates in fatty acid metabolism, such as the third step of β-oxidation^50^. AMP-binding enzyme (Os01g46750) is directly responsible for catalyzing de novo purine synthesis. Apical meristem protein (Os05g34310) is involved in nucleotide and nucleic acid metabolic processes. These proteins are related to fatty acid metabolism, amino acid biosynthesis and nucleotide metabolism, respectively. ER stress can influence metabolism and cause metabolic disorder, meanwhile, some metabolic signal pathways in response to relieve the stress by regulating related genes expression^51^, which may be the reason of these proteins with altered expression under ER stress. Besides above mentioned, there are 6 proteins can also be classified as metabolic proteins (Os03g61130, Os02g17390, Os01g56810, Os04g32560, Os12g38900, Os05g08750), but the functional roles of these proteins are still unknown. Future research of these proteins will further enhance our understanding of the relationship between ER stress signal and other metabolic pathways^52^.

ER stress responses are involved in both abiotic and biotic stress in plants. In *OsSar1* transgenic seeds, 16 upregulated proteins were categorized as stress and defense response proteins. Six proteins (Os10g35720, ChrSy.fgenesh.gene.69, Os04g41960, Os07g34570, Os02g02400 and Os08g43560) were putatively classified as involved in redox homeostasis. However, a  2.0- fold decrease in the expression of 6 oxidoreductase proteins was found (Os07g08840, Os01g43090, Os05g38230, Os05g39690, Os05g04870 and Os07g08950) (Table S10). Cellular redox homeostasis plays a key role in mediating various physiological and developmental processes, which comprise many factors. Alterations in the ER redox state can result in ER stress, and can induce a set of proteins involved in protein folding in the ER^53^. In addition to the 16 upregulated proteins, 65 stress- and defense-related proteins were downregulated (Table S10).

In addition to these metabolism-, stress- and defense-related proteins, 2 glycosyl hydrolases (Os10g28120 and Os08g13920), 4 stress-associated proteins (Os07g47510, Os12g19381, Os12g17600 and Os04g32330), an actin-7 protein (Os01g64630), a leaf bladeless1 (Os12g09580), a late embryogenesis abundant protein (Os03g62620) and a PAP fibrillin family domain-containing protein (Os09g04790) were upregulated. These proteins play diverse roles in various stress responses. For example, glycosyl hydrolases are involved in a diverse range of processes, including starch metabolism, defense and cell wall remodeling^54^. The proteins that are abundant during late embryogenesis are involved in drought-, cold- and salinity-stress tolerance in plants^55^.

### Proteins downregulated in seed of *OsSar1* RNAi rice

Compared with the WT control, 265 proteins were downregulated ≥2.0-fold in the *OsSar1* transgenic seeds. These proteins were classified into 9 groups by function, including stress and defense proteins (24.5%), carbohydrate metabolic processes (12.8%), protein metabolic processes (8.7%), protein folding and modification proteins (8.3%), transport proteins (5.3%), cell redox homeostasis (2.3%), signal transducer (0.7%), other metabolism-related proteins (26.8%), and unknown/others (10.6%) (Fig. 3B and Table S10). A > 5- fold decrease in expression of 59 proteins was found, including Sar1c (Table S11). In general, lipid transfer proteins (LTPs) and the HSP20/alpha crystallin family proteins were downregulated.

LTPs facilitate lipid transport between membranes, and they play an important role in various plant physiological processes, including cutin synthesis, β-oxidation, somatic embryogenesis, defense reactions, signal transduction and responses to various environmental conditions^56^. The expression of 10 LIPs significantly decreased. Thus, the suppression of *Sar1* disturbed protein transport but also may have affected lipid transport.

Hsp20 represents the most abundant small heat shock proteins (sHSPs) in plants^56^. HSP20 is required in a common developmental route in seeds. Oono *et al.* reported that the HSP20 proteins expression differed among the development stages, but was not upregulated after ER stress in rice. We found that the expression of 5 HSP20 family proteins was decreased, which indicated that abnormal seed development may affect the expression of the HSP20 family proteins.

### Verification of iTRAQ data with selected candidates by quantitative real-time PCR (qPCR) and Western blot

**Figure 4:**
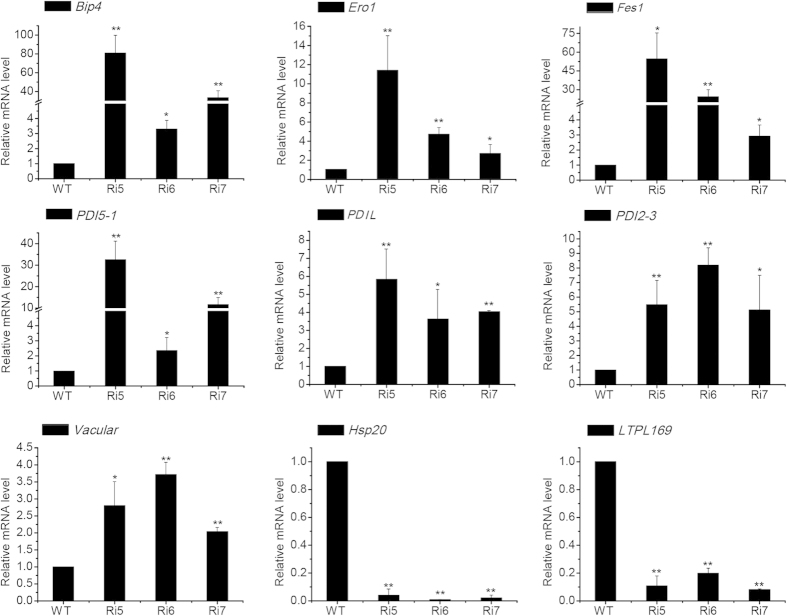
qPCR analysis of the mRNA expression of genes that correspond to differentially expressed proteins. Relative mRNA expression levels of 9 differentially expressed proteins in *OsSar1* RNAi transgenic rice. Values are the mean ± SD (standard deviation) of 3 independent qPCR experiments. *p < 0.05, **p < 0.01, and ***p < 0.001 versus wild type group (Student’s t-test).

We used qPCR to analyze the gene expression profiles of a subset of proteins that are specifically induced or suppressed in these transgenic seeds. The 9 representative genes were *BiP4* (Os05g35400), *Ero1* (Os03g52340), *Fes1* (Os09g33780), *PDI5–1* (Os03g17860), *PDIL* (Os03g29240), *PDI2–3* (Os09g27830), *Vacular* (Os10g35250), *Hsp20* (Os03g15960) and *LTPL169* (Os07g12080). The levels of *BiP4*, *Fes1* and *PDI5–1* increased more than 30-fold in the transgenic lines compared with the WT control (Fig. 4). *Ero1*, *PDIL*, *PDIL2–3* and *Vacular* were significantly upregulated and *HSP20* and *LTPL169* were downregulated in transgenic seeds. These results were consistent with the iTRAQ assay data. We examined the expression of these genes in DTT- or tunicamycin-induced ER-stressed seedlings by qPCR, and the results of this analysis were similar to those obtained in ER-stressed seeds (Fig. S1). In addition, the expression level of another two proteins (OsBip4&5 and LOC_Os01g03360) was detected by Western blot (Fig. S2), which was consistent with our iTRAQ data. For example, as previously reported, the expression of OsBip4&5 was not detected under normal conditions in rice, but highly and specifically activated under ER stress conditions^28^. Therefore, the differential expression of the proteins identified by iTRAQ assay was due to ER stress.

### Identification of metabolic pathways significantly upregulated by ER stress

**Figure 5:**
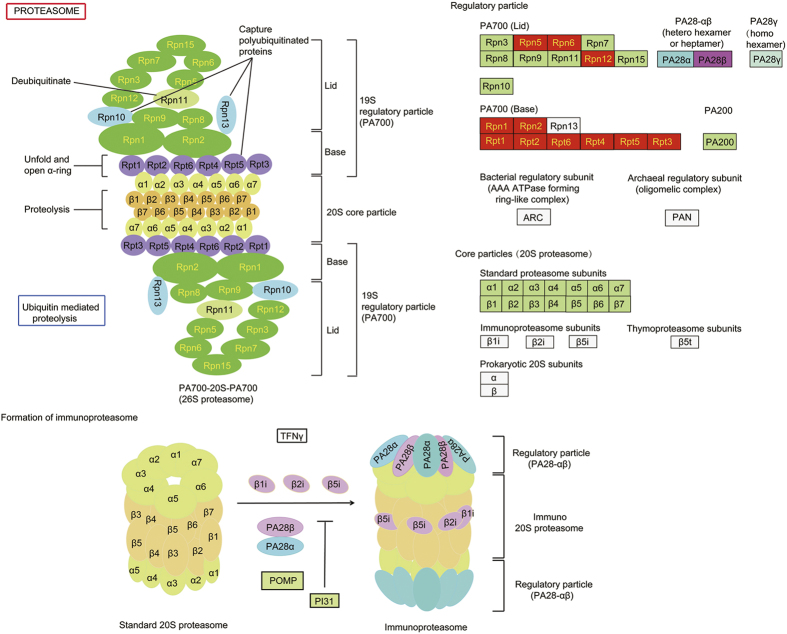
Upregulated proteins in the proteasome pathway. Pathway analysis performed with KOBAS 2.0. Red boxes are proteins that were found to be upregulated by the iTRAQ assay.

**Figure 6:**
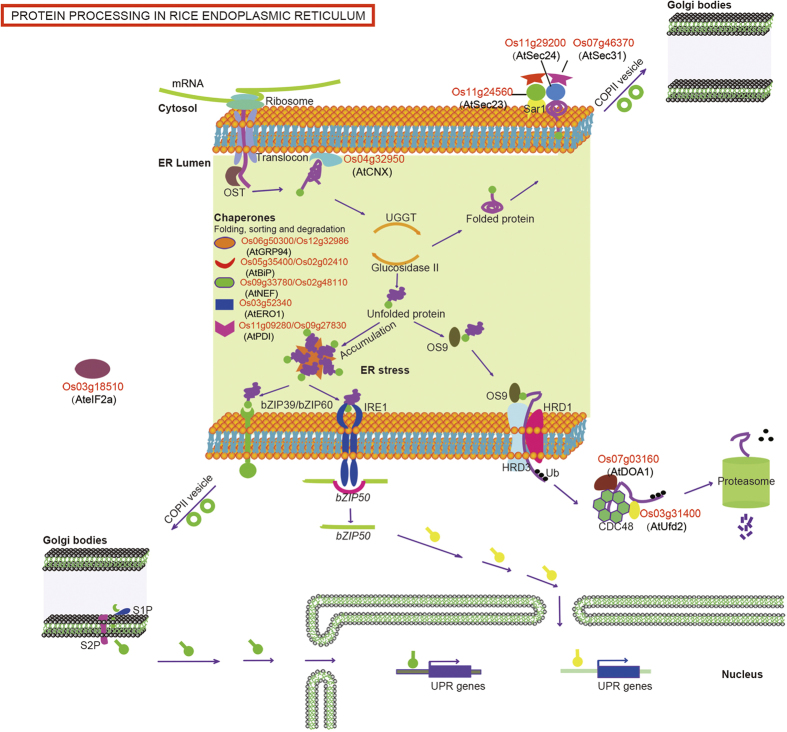
Upregulated proteins in rice ER protein-processing pathway. Schematic illustration of the core elements of rice specific protein-processing pathway and UPR signaling network. Proteins labled in red indicate that the upregulated proteins by the iTRAQ assay, whose orthologous in *Arabidopsis* were labled in bracket. Chaperones that have diversity functions were listed out briefly.

To gain insight into the upregulated biochemical reactions during ER stress in rice seeds, we used KOBAS 2.0 and identified 45 significantly upregulated pathways. Seven of these pathways were significantly upregulated in response to ER stress (p < 0.05), based on hypergeometric distribution. Two of these pathways had p value < 0.01 after the FDR correction (Table S12) with 11 and 16 upregulated proteins involved in the proteasome pathway and in protein processing in the ER, respectively (Figs 5 and 6). The 11 upregulated proteins involved in the proteasome pathway were 7 26S protease regulatory subunits (Os02g10640, Os07g49150, Os02g56000, Os02g21970, Os02g11050, Os02g54340 and Os07g25420), 2 proteasome/cyclosome repeat-containing proteins (Os02g05340 and Os08g12820) and 2 proteasome subunits (Os04g36700 and Os03g63430) (Fig. 5). The 16 upregulated proteins involved in protein-processing in the ER were a translation initiation factor (Os03g18510), BiP1 (Os02g02410), BiP4 (Os05g35400), PDIL1-1 (Os11g09280), PDIL2-3 (Os09g27830), Ero1 (Os03g52340), Calnexin (Os04g32950), a DnaK family protein (Os02g48110), a Fes1-like protein (Os09g33780), a U-box domain-containing protein (Os03g31400), 2 HSPs (Os12g32986 and Os06g50300), 2 protein transporters (Os11g24560 and Os11g29200) and 2 WD domain, G-beta repeat domain-containing proteins (Os07g03160 and Os07g46370) (Fig. 6). The most of these proteins are chaperons that facilitate folding, sorting, or degradation, relieving the stress of ER through upregulated their expression. Interestingly, a translation initiation factor Os03g18510 (AteIF2a) was upregulated, does translation rate in response to ER stress? Are PERK-like proteins functional in plant UPR? Is there a third arm of the UPR pathway in plants? Further studies will be needed to answer these major unsolved question in plant UPR.

## Methods

### Generation of transgenic plants

Seed-specific ER-stressed transgenic rice plants were generated by suppressing *OsSar1a/b/c* using RNAi as previously described. Successful transformants were verified by PCR and grown in a greenhouse. Developing seeds at 10 to 15 DAF were used for the iTRAQ analysis.

### SDS-PAGE and Western blot analysis

Rice seeds were ground to a powder, and total protein was extracted with buffer (0.125 M Tris-Cl, 4 M urea, 4% SDS and 2% β-mercaptoethanol, pH 6.8). The proteins were fractioned by SDS-PAGE and transferred to a PVDF membrane (Millipore, USA). A Western blot analysis was performed as previously described.

### Protein sample preparation

Low salt-soluble proteins were extracted by grinding developing rice seeds in 800 μl of extraction buffer that contained 10 mM HEPES (pH 6.8), 20 mM NaCl, and 10 mM PMSF, followed by 3 min of ultrasonic treatment in an ice-water bath. The homogenate was centrifuged at 14000 × *g* for 15 min at 4 °C. The supernatant was collected to a new centrifuge tube, and the previous step was repeated. The total supernatant was then purified by acetone precipitation. The resulting pellets were collected and resolved with 4 M urea, and the total protein was quantified using the 2-D Quant Kit (GE Healthcare).

### Trypsin digestion

The iTRAQ 4-plex kit (Applied Biosystems, CA) was used according to the manufacturer’s instructions, with slight modifications. Protein extracts (100 μg each) were added to 1 μl of denaturant and reducing reagent and then heated at 37 °C for 1 hr. The samples were spun and incubated at room temperature for 10 min with 1 μl of cysteine blocking reagent. The protein samples buffer was replaced with dissolution buffer using the Filter-Aided Sample Preparation method, and the sample was incubated overnight with 2 μg of trypsin at 37 °C.

### iTRAQ labelling

iTRAQ reagents are non-polymeric, isobaric tagging reagents consisting of a peptide reactive group, a reporter group, and a balance group. Our iTRAQ experiment included 4-plex labeling, which was accomplished by first adding 150 μl of ethanol was added to the iTRAQ reagent. iTRAQ reagents 114 and 116 were then transferred to the control samples, and iTRAQ reagents 115 and 117 were then added to the treated samples. All samples were then incubated at room temperature for 2 hrs. The reaction was quenched by adding 100 μl of Milli-Q water to each tube at room temperature for 1 hr. The iTRAQ-labelled samples were combined into one tube, dried in a vacuum centrifuge and stored at −80 °C. Two biological replicates were performed.

### High-pH reverse-phase liquid chromatography

High-pH reverse-phase chromatography with a C18 column (Durashell-C18, 4.6 mm × 250 mm, 5 μm, 100 Å, Agela) was used to label the iTRAQ peptide samples. The column was equilibrated with 20 mM ammonium formate (pH 10), which was also used to resuspend the samples. The peptides were eluted with a 65 min gradient to 100% 20 mM ammonium formate (pH 10) in 80% v/v acetonitrile (ACN), at a flow rate of 0.8 mL/min. The peptides were separated into 5 fractions for the LC-MS/MS analysis.

### iTRAQ nanoflow LC-MS/MS

Each fraction was resuspended in 30 μl of 0.1% v/v formic acid (FA) and 10 μl was used with an Eksigent NanoLC Ultra system (AB SCIEX). The samples were desalted on a 100 μm × 20 mm trap column and eluted onto a 75 μm × 150 mm analytical column. Both the trap and the analytical columns were filled with MAGIC C18AQ, 5 μm, 200 Å phase (michrom BIORESOURCES, Inc.). The peptides were separated by a gradient formed by 0.1% FA (mobile phase A) and 100% ACN, 0.1% FA (mobile phase B), from 5% to 30% of the mobile phase B in 75 min at a flow rate of 0.3 μl/min. The MS analysis was performed on a TripleTOF 5600 system (AB SCIEX) in Information-Dependent Mode. The MS spectra were acquired across a mass range of 350–1500 m/z in high-resolution mode (>30,000) with a 250- ms accumulation time per spectrum. A maximum of 40 precursors per cycle was selected for fragmentation from each MS spectrum with a 100- ms minimum accumulation time for each precursor and dynamic exclusion for 20 s. Tandem mass spectra were recorded in high-sensitivity mode (resolution > 15,000) with rolling collision energy.

### iTRAQ data analysis

The peak lists for the MS/MS spectra were processed by ProteinPilot v4.5 (AB SCIEX) to identify the proteins, and then quantified using the Paragon algorithm (AB SCIEX). The data were searched against the NCBI *O. sativa* database downloaded on May 1, 2013. The following Paragon algorithm parameters were used: the sample type was iTRAQ 4-plex (peptide labelled); the cysteine alkylation was methyl methane-thiosulfonate (MMTS); the digestion was trypsin specificity; the search effort was a thorough ID; and the processing was background, quantitation and bias correction. The proteins were identified as having at least two distinct peptides with a 99% confidence and a 2.0 contribution to the unused ProtScore, which created an even more reliable protein list. Proteins with an Unused ProtScore ≥4.0 were included in the final protein list. The threshold for the reliable quantitation of protein expression was set at p < 0.05.

### Functional classification by gene ontology

The gene ontology information for the functional classification of identified proteins used blast2go (http://www.blast2go.com/b2ghome?eprivacy=1) and the Rice Genome Annotation Project (http://rice.plantbiology.msu.edu/index.shtml). The protein pathway analysis was performed using KOBAS 2.0.

### qPCR analysis

*Oryza sativa cv. Kitaake* plants were grown on MS medium that contained with 0.4% Gelrite for eight days at 28 °C using a 16 h/8 h light/dark cycle. Subsequently, the seedlings were incubated for 4 h in liquid MS medium that contained 5 μg/ml of tunicamycin (Tm) and 2 mM DTT. Equal volumes of DMSO (final concentration of 0.1%) and water were added as the negative controls for Tm and DTT, respectively.

Total RNA was extracted from developing seeds at 10 DAF using TRIpure Reagent (Bioteke Corp.). RNA was reverse-transcribed with use of the PrimeScript II 1^st^ strand cDNA Synthesis Kit (Takara). qPCR was performed on LightCycler system (Roche Diagnostics). The primers were designed by Beacon Designer 8.0 (Supplementary Table S1). The gene expression was normalized to that of rice *Act-1* (GeneBank accession no.: X16280) as an internal control. The data represent the mean of 3 independent experiments.

### Statistical Analysis

The qPCR values are presented as the mean ± S.D. A statistical analysis was performed using Student’s t test to evaluate significant differences, which were expressed using a p value. p values lower than 0.05 were considered to indicate significant difference. The varying degrees of significance were p < 0.05, p < 0.01, and p < 0.001, and were labled with one asterisk (*), two asterisks (**), and three asterisks (***), respectively.

## Additional Information

**How to cite this article**: Qian, D. *et al.* Proteomic analysis of endoplasmic reticulum stress responses in rice seeds. *Sci. Rep.* **5**, 14255; doi: 10.1038/srep14255 (2015).

## Supplementary Material

Supplementary data

Table S2

Table S3

Table S4

Table S5

Table S6

Table S7

## Figures and Tables

**Table 1:** List of proteins increased in *OsSar1* RNAi transgenic rice more than 2.0-folds*.

Accession no.^a^	Foldchange^b^	Orthologous^c^	Protein description	Loc no.^d^
Protein translation				
gi|222635978	2.323	AT4G10320	tRNA synthetase class I	LOC_Os06g43760
gi|222630224	3.842	AT3G02760	tRNA synthetase class II core domain containing protein	LOC_Os05g05840
gi|218184221	2.516	AT1G50200	alanyl-tRNA synthetase	LOC_Os10g10244
gi|109940177	2.058	AT4G13780	methionyl-tRNA synthetase	LOC_Os06g31210
gi|115462445	2.088	AT1G25350	glutaminyl-tRNA synthetase	LOC_Os05g08990
gi|218197256	2.454	AT1G72550	phenylalanyl-tRNA synthetase beta chain	LOC_Os05g48510
gi|222637260	2.004	AT3G60240	eukaryotic translation initiation factor 4G	LOC_Os07g36940
gi|83306210	2.806	AT2G40290	Translation initiation factor 2	LOC_Os03g18510
gi|125544262	3.768	—	elongation factor protein	LOC_Os03g29260
gi|115456914	2.635	AT1G56070	elongation factor	LOC_Os04g02820
gi|297597332	7.757	AT2G34480	60S ribosomal protein L18a	LOC_Os01g47660
gi|2058273	4.159	AT1G33120	ribosomal protein L6	LOC_Os09g31180
gi|303844	3.158	—	DEAD-box ATP-dependent RNA helicase	LOC_Os06g48750
Protein folding and modification				
gi|115443791	5.226	AT5G28540	BiP1	LOC_Os02g02410
gi|115464027	27.984	—	BiP4	LOC_Os05g35400
gi|297727109	3.566	—	DnaK family protein	LOC_Os09g31486
gi|115448237	3.706	AT4G16660	DnaK family protein	LOC_Os02g48110
gi|222617173	2.656	AT3G07770	Heat shock protein	LOC_Os12g32986
gi|18855040	10.001	AT4G24190	Heat shock protein	LOC_Os06g50300
gi|297607496	2.847	AT5G19820	HEAT repeat family protein	LOC_Os07g38760
gi|115478158	3.160	AT1G13320	HEAT repeat family protein	LOC_Os09g07510
gi|115444457	3.557	AT2G02560	HEAT repeat family protein	LOC_Os02g07120
gi|385717664	2.055	AT1G21750	OsPDIL1-1protein disulfide isomerase PDIL1-1	LOC_Os11g09280
gi|115479475	6.142	AT1G04980	OsPDIL2-3 protein disulfide isomerase PDIL2-3	LOC_Os09g27830
gi|125585868	10.486	AT1G07960	OsPDIL5-1 protein disulfide isomerase PDIL5-1	LOC_Os03g17860
gi|75327654	3.036	AT1G60420	PDI	LOC_Os03g29240
gi|29150368	4.928	—	Oxidoreductase	LOC_Os03g58880
gi|115458184	3.199	AT5G07340	Calnexin	LOC_Os04g32950
gi|50582733	6.742	AT1G72280	ERO1	LOC_Os03g52340
gi|218197595	4.374	AT4G29520	expressed protein	LOC_Os06g05740
gi|45272584	5.510	AT1G48600	CPuORF25—conserved peptide uORF-containing transcript	LOC_Os01g50030
gi|115468554	2.197	AT1G24510	T-complex protein	LOC_Os06g36700
Protein degradation				
gi|115444877	2.723	AT1G45000	26S protease regulatory subunit	LOC_Os02g10640
gi|115474241	2.457	AT2G20140	26S protease regulatory subunit 4	LOC_Os07g49150
gi|556560	4.396	—	26S protease regulatory subunit 6A	LOC_Os02g56000
gi|115445841	3.909	AT5G58290	26S protease regulatory subunit 6B	LOC_Os02g21970
gi|115444937	6.237	AT5G19990	26S proteasome regulatory particle triple-A ATPase subunit6	LOC_Os02g11050
gi|125596332	2.299	AT1G53750	26S protease regulatory subunit 7	LOC_Os02g54340
gi|218199510	5.739	AT1G64520	proteasome regulatory particle	LOC_Os07g25420
gi|222622165	2.796	AT2G20580	proteasome/cyclosome repeat containing protein	LOC_Os02g05340
gi|125591574	3.300	AT1G04810	proteasome/cyclosome repeat containing protein	LOC_Os08g12820
gi|115458588	3.393	AT1G29150	proteasome subunit	LOC_Os04g36700
gi|56783671	3.395	AT2G26990	proteasome subunit	LOC_Os01g17180
gi|218194124	4.286	AT5G64760	proteasome subunit	LOC_Os03g63430
gi|32489165	2.487	AT3G02200	proteasome subunit	LOC_Os04g01290
gi|50509991	2.210	AT5G42220	ubiquitin family protein	LOC_Os07g31540
gi|115482252	4.019	AT1G63800	ubiquitin-conjugating enzyme	LOC_Os10g31000
gi|115474297	3.156	AT3G55410	2-oxoglutarate dehydrogenase E1 component	LOC_Os07g49520
gi|75261364	4.276	AT2G24200	leucine aminopeptidase	LOC_Os02g55140
gi|311893431	6.100	AT4G33150	saccharopine dehydrogenase	LOC_Os02g54254
gi|222616995	4.201	AT1G55860	HECT-domain domain containing protein	LOC_Os12g24080
gi|37718894	2.670	AT5G15400	U-box domain-containing protein	LOC_Os03g31400
gi|125987818	7.325	—	Cysteine proteinase inhibitor 2	LOC_Os05g41460
gi|297720695	3.595	AT2G31980	cysteine proteinase inhibitor precursor	LOC_Os01g68670
gi|125551937	2.052	AT3G51800	peptidase, M24 family protein	LOC_Os05g28280
gi|115480101	25.975	AT3G51980	fes1-like protein	LOC_Os09g33780
Transport proteins				
gi|642121	3.005	AT4G17170	ras-related protein	LOC_Os02g37420
gi|62734287	2.081	AT1G05520	protein transport protein	LOC_Os11g24560
gi|77550927	2.377	AT3G44340	protein transport protein	LOC_Os11g29200
gi|115463365	4.855	AT3G08943	importin subunit beta	LOC_Os05g28510
gi|7339699	2.312	AT2G46520	importin-alpha re-exporter	LOC_Os01g13430
gi|6682927	2.261	AT3G06720	importin subunit alpha	LOC_Os05g06350
gi|125578219	3.519	AT3G08530	clathrin heavy chain	LOC_Os12g01390
gi|125578212	3.461	AT3G08530	clathrin heavy chain	LOC_Os11g01380
gi|115446279	3.129	AT1G05350	ThiF family domain containing protein	LOC_Os02g30310
gi|115478689	6.321	AT4G27500	BRI1-KD interacting protein 103	LOC_Os09g17730
gi|222637015	3.653	AT5G66030	ATGRIP/GRIP	LOC_Os07g28940
gi|29367377	2.750	AT1G12840	vacuolar ATP synthase subunit C	LOC_Os05g51530
gi|125532495	7.786	AT1G15130	vacuolar protein-sorting protein bro1	LOC_Os10g35250
gi|115456717	4.368	—	retrotransposon protein	LOC_Os03g64080
gi|115452177	2.074	AT3G46740	outer membrane protein	LOC_Os03g16440
gi|115444901	3.237	—	mitochondrial carrier protein	LOC_Os02g10800
Signal transducer				
gi|115476520	3.527	AT3G02520	14-3-3 protein	LOC_Os08g33370
gi|115454901	3.359	AT1G22300	14-3-3 protein	LOC_Os03g50290
gi|115465831	2.269	AT4G11010	nucleoside diphosphate kinase	LOC_Os05g51700
gi|27497122	2.975	AT5G21090	BRASSINOSTEROID INSENSITIVE 1-associated receptor kinase 1 precursor	LOC_Os01g59440
gi|218199020	2.451	AT3G18860	WD domain, G-beta repeat domain containing protein	LOC_Os07g03160
gi|222637603	3.945	AT1G18830	WD domain, G-beta repeat domain containing protein	LOC_Os07g46370
gi|115454489	2.501	AT2G30880	pleckstrin homology domain-containing protein	LOC_Os03g46340
gi|115436494	2.157	—	AIG1 family protein	LOC_Os01g25450
Metabolism				
gi|115451283	2.754	AT2G22240	inositol-3-phosphate synthase	LOC_Os03g09250
gi|125557458	2.382	AT3G54640	indole-3-glycerol phosphate lyase	LOC_Os07g08430
gi|75138360	2.637	AT1G22410	phospho-2-dehydro-3-deoxyheptonate aldolase	LOC_Os07g42960
gi|115468200	3.833	AT1G10760	alpha-glucan water dikinase	LOC_Os06g30310
gi|125544232	2.056	AT3G43190	sucrose synthase	LOC_Os03g28330
gi|34015340	2.317	AT1G04920	sucrose-phosphate synthase	LOC_Os08g20660
gi|218196223	7.542	—	hexokinase	LOC_Os05g09500
gi|115463815	3.416	AT4G15530	pyruvate, phosphate dikinase	LOC_Os05g33570
gi|115465825	13.676	AT4G10960	UDP-glucose 4-epimerase 1	LOC_Os05g51670
gi|32490267	2.354	AT2G43180	carboxyvinyl-carboxyphosphonate phosphorylmutase	LOC_Os04g42580
gi|115456435	2.048	AT1G50940	electron transfer flavoprotein subunit alpha	LOC_Os03g61920
gi|115480417	3.038	AT5G66120	3-dehydroquinate synthase	LOC_Os09g36800
gi|115436430	2.815	AT3G06860	3-hydroxyacyl-CoA dehydrogenase	LOC_Os01g24680
gi|218188780	2.461	AT4G11030	AMP-binding enzyme	LOC_Os01g46750
gi|213959137	3.561	—	no apical meristem protein	LOC_Os05g34310
gi|15042826	2.602	AT1G07230	phosphoesterase family protein	LOC_Os03g61130
gi|115445513	4.220	AT4G29010	3-hydroxyacyl-CoA dehydrogenase	LOC_Os02g17390
gi|125572210	2.680	AT1G75450	cytokinin dehydrogenase precursor	LOC_Os01g56810
gi|125590233	3.409	AT3G48870	ATP-dependent Clp protease ATP-binding subunit clpA homolog CD4B	LOC_Os04g32560
gi|115489266	3.613	—	chorismate mutase	LOC_Os12g38900
gi|297723761	3.062	—	UDP-glucoronosyl and UDP-glucosyl transferase domain containing protein	LOC_Os05g08750
gi|20208	3.771	AT1G03880	Glutelin	LOC_Os03g31360
gi|76564691	6.413	AT1G03880	glutelin	LOC_Os10g26060
gi|428674406	7.838	AT1G03880	glutelin, partial	LOC_Os02g15090
gi|115445465	5.442	AT1G03880	Glutelin type-B 5	LOC_Os02g16820
Stress and defense				
gi|122063509	2.774	AT4G04950	OsGrx_S17 - glutaredoxin subgroup II	LOC_Os10g35720
gi|222628934	4.969	—	Putative 12-oxophytodienoate reductase 12	ChrSy.fgenesh.gene.69
gi|115459206	2.324	AT1G26320	NADP-dependent oxidoreductase	LOC_Os04g41960
gi|27261025	2.893	AT5G54770	FAD dependent oxidoreductase domain containing protein	LOC_Os07g34570
gi|283050393	3.120	—	catalase isozyme A	LOC_Os02g02400
gi|115477687	3.566	AT4G35000	OsAPx4 - Peroxisomal Ascorbate Peroxidase encoding gene 5,8,9	LOC_Os08g43560
gi|115482030	4.913	—	glycosyl hydrolase	LOC_Os10g28120
gi|125572628	4.696	—	glycosyl hydrolases family 16	LOC_Os08g13920
gi|22831130	2.240	AT2G47780	stress-related protein	LOC_Os07g47510
gi|149392260	3.883	AT1G67090	ribulose bisphosphate carboxylase small chain	LOC_Os12g19381
gi|347451	4.176	AT1G67090	ribulose bisphosphate carboxylase small chain	LOC_Os12g17600
gi|115458104	3.492	AT4G26910	unnamed protein product	LOC_Os04g32330
gi|148886771	2.377	—	actin	LOC_Os01g64630
gi|125578786	6.226	AT5G23570	leafbladeless1	LOC_Os12g09580
gi|125588600	3.082	AT2G44060	late embryogenesis abundant protein	LOC_Os03g62620
gi|62900682	8.878	AT4G04020	PAP fibrillin family domain containing protein	LOC_Os09g04790
RNA binding protein				
gi|115446411	5.570	AT5G07350	RNA binding protein Rp120	LOC_Os02g32350
gi|125572738	3.988	AT4G32720	RNA binding protein	LOC_Os04g42010
gi|115449135	2.466	AT3G11400	RNA recognition motif containing protein	LOC_Os02g54700
Unknown/others				
gi|115439385	2.780	—	VIP1 protein	LOC_Os01g50310
gi|115444621	2.953	AT2G24020	CR084 protein	LOC_Os02g08380
gi|34394390	2.208	AT5G42950	GYF domain containing protein	LOC_Os07g04530
gi|115459588	4.002	AT1G63220	C2 domain containing protein	LOC_Os04g44870
gi|77552025	6.062	AT2G22660	DUF1399 containing protein	LOC_Os11g40590
gi|116309939	4.359	—	MA3 domain containing protein	LOC_Os04g40660
gi|115473741	10.989	AT1G03350	BSD domain-containing protein	LOC_Os07g45310
gi|125604008	2.370	AT5G25070	uvrB/uvrC motif family protein	LOC_Os08g40450
gi|38345301	2.604	AT1G14570	UBX domain-containing protein	LOC_Os04g57520
gi|125532667	2.843	AT4G10790	UBX domain-containing protein	LOC_Os10g37630
gi|77555438	2.321	AT5G13200	GRAM domain containing protein	LOC_Os12g29400
gi|218196211	2.353	AT4G39470	chloroplast lumen common family protein	LOC_Os05g08930
gi|115448305	2.726	AT3G52140	tetratricopeptide repeat containing protein	LOC_Os02g48620
gi|17027265	2.375	AT1G49520	upstream activation factor subunit spp27	LOC_Os03g55570
gi|115470389	2.382	AT4G37210	tetratricopeptide repeat containing protein	LOC_Os07g03070

^*^P ≤ 0.05.

^a^Accession no. is the name of a gene in the NCBI (National Center for Biotechnology Information).

^b^The values were calculated as the average ratio of 115, 117 (*OsSar1* RNAi) to 114, 116 (kitaake) label.

^c^Orthologous is the name of homologue of *Arabidopsis* according to the RGAP (Rice Genome Annotation Project Database).

^d^Loc no. is the locus name of a gene in the RGAP (Rice Genome Annotation Project Database).
