# Supplementary material for: Proteomic analysis of endoplasmic reticulum stress responses in rice seeds
Source: Sci Rep. 2015 Sep 23;5:14255. doi: 10.1038/srep14255 (PMC4585792; doi:10.1038/srep14255)
Supplement: Supplementary data [file srep14255-s1.doc]

**Supplemental online data**

**Proteomic analysis of endoplasmic reticulum stress responses in rice seeds**

Dandan Qian, Lihong Tian, Leqing Qu*

Key Laboratory of Plant Molecular Physiology, Institute of Botany, the Chinese Academy of Sciences, Beijing 100093, China

**Figure S1:** qRT-PCR analysis of 9 differential expressed proteins in rice seedlings treated with endoplasmic-reticulum stress inducers. The values are mean ± SD (standard deviation) of 3 independent qRT-PCR experiments. *p <0.05, and **p <0.01 (Student's t-test), DTT and Tm versus H_2_O and DMSO, respectively.

**Figure S2:** Western blot analysis of 2 differently expressed proteins in iTRAQ

experiment.

**Table S1:** Oligonucleotide primers were used for qPCR.

**Table S2:** The Protein ID Summary of the first independent experiment

**Table S3:** The Protein ID Summary of the technical duplicates

**Table S4:** The Differential Protein Summary of the first biological experiment

**Table S5:** The Differential Protein Summary of the first biological replicates

**Table S6:** The Differential Protein Summary of the second biological experiment

**Table S7:** The Differential Protein Summary of the second biological replicates

**Table S8:** Summary of proteins identified at critical false discovery rate (FDR) in the first independent experiment.

**Table S9:** Summary of proteins identified at critical FDR in the second independent experiment.

**Table S10:** Proteins downregulated by > 2.0-fold in *OsSar1* transgenic rice.

**Table S11:** Proteins downregulated by > 5.0-fold in *OsSar1* transgenic rice.

**Table S12:** Protein upregulated preferential metabolic pathways were identified by KOBAS.

**Supplementary Figure S1**

**
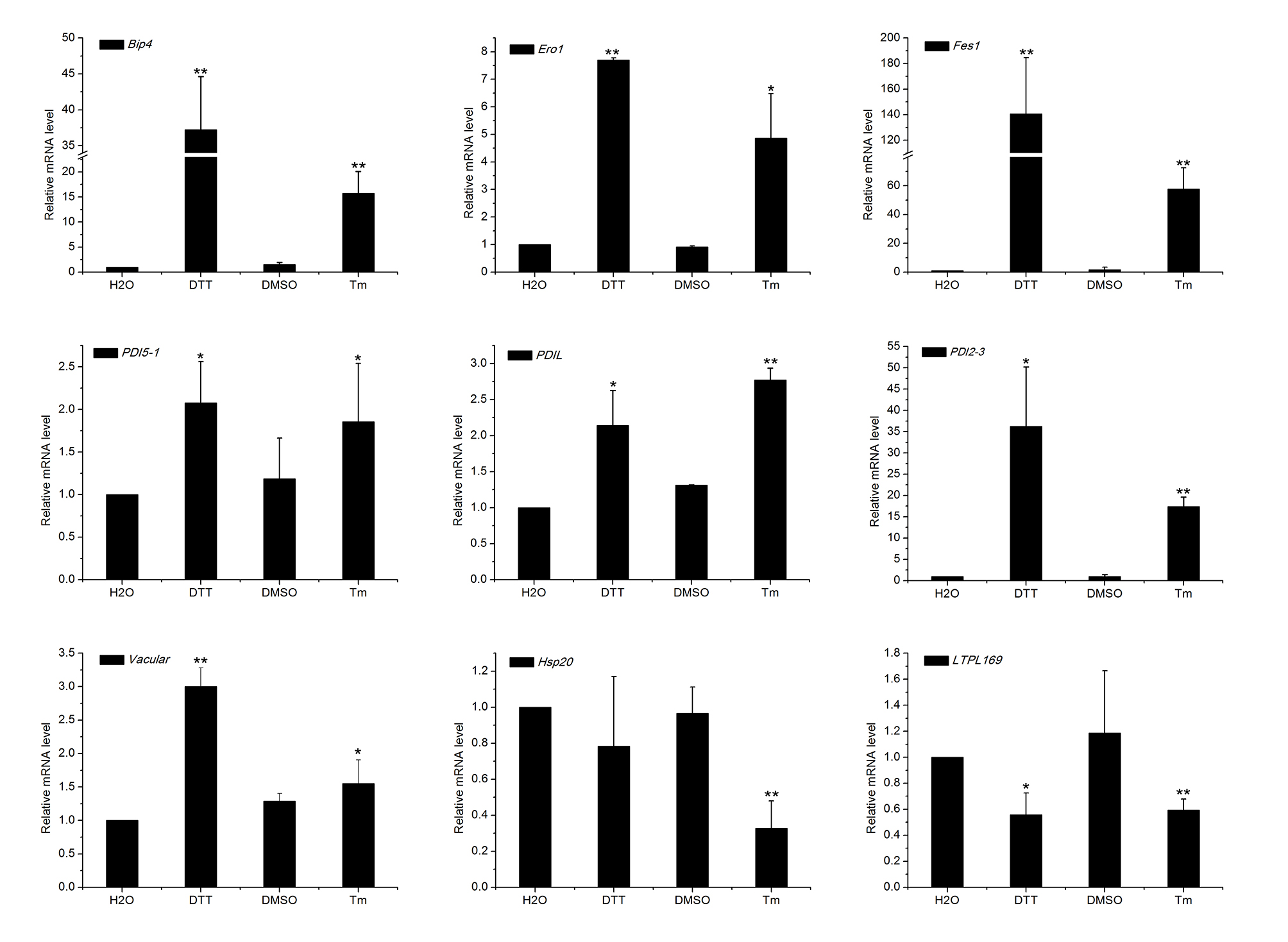
**

**Supplementary Figure S2**

**
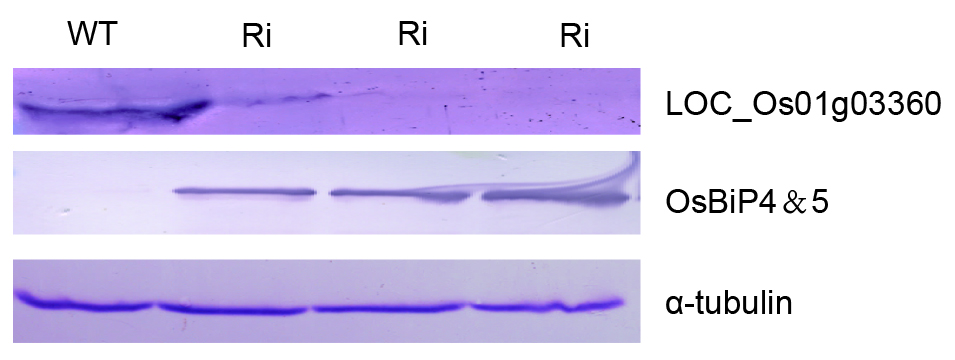
**

**Supplementary Table S1.** Oligonucleotide primers were used for qPCR.

| Primer Names^a^ | Sequences |
| --- | --- |
| BiP4-F | 5’- AGAGCATGATCCTCCTCGAC - 3’ |
| BIP4-R | 5’- GTCGTCTGCCTGTCCTTGTA - 3’ |
| ERO1-F | 5’- TCGCAGAATATGGGACTCTG - 3’ |
| ERO1-R | 5’- GAGGAGTGCAACCCTGAAAT - 3’ |
| fes1-like-F | 5’- CGTGCACTGCAGGAACTATT - 3’ |
| fes1-like-R | 5’- CCATGCAGAAGTGGTCCTTA - 3’ |
| OsPDIL5-1-F | 5’-TTTGTGAAGTTCTGCGTTCC - 3’ |
| OsPDIL5-1-R | 5’-TCCACCTTTGAGCATACTGG - 3’ |
| PDI-F | 5’-TTGATGAGCATGGTCCAGAT - 3’ |
| PDI-R | 5’-GGACTCCAGGGTCTGTGATT - 3’ |
| OsPDIL2-3-F | 5’-CCTGGAAAGCCTCCAGTAGA - 3’ |
| OsPDIL2-3-R | 5’- CTGATTTCTTGCCACCTGAA - 3’ |
| Vacuolar-F | 5’- CCATGACTATGGCCAACAAG - 3’ |
| Vacuolar-R | 5’- TGGTGAGGAGGGTAAGAACC - 3’ |
| hsp20-F | 5’- GATCGACTGGAAGGAGACG - 3’ |
| hsp20-R | 5’-TTCTCCTCCTGCTCCTTGTT - 3’ |
| LTPL169-F | 5’-GTGCAACCTCGAGACCATC - 3’ |
| LTPL169-R | 5’-CAATATTTGGGCATCTCCCT - 3’ |

^a^ Abbreviations: F, forward primer; R, reverse primer.

**Supplementary Table S8:** Summary of proteins identified at critical false discovery rate (FDR) in the first independent experiment.

|  | Number of Proteins Detected | | |
| --- | --- | --- | --- |
| Critical FDR | Local FDR | Global FDR | Global FDR from Fit |
| 1.0% | 2971 | 3272 | 3277 |
| 5.0% | 3099 | 3525 | 3541 |
| 10.0% | 3155 | 3762 | 3819 |

**Supplementary Table S9:** Summary of proteins identified at critical FDR in the second independent experiment.

|  | Number of Proteins Detected | | |
| --- | --- | --- | --- |
| Critical FDR | Local FDR | Global FDR | Global FDR from Fit |
| 1.0% | 2976 | 3292 | 3293 |
| 5.0% | 3113 | 3544 | 3556 |
| 10.0% | 3170 | 3826 | 3820 |

**Supplementary Table S10:** Proteins downregulated by > 2.0-fold in *OsSar1* transgenic rice.

| **Accession no.** | **Fold change** | **Orthologous** | **Protein description** | **Loc no.** |
| --- | --- | --- | --- | --- |
| **Transport proteins** | | | | |
| gi|115435714 | 0.166 | - | sar1c | LOC_Os01g15010 |
| gi|115474931 | 0.464 | AT1G31812 | acyl CoA binding protein | LOC_Os08g06550 |
| gi|55296316 | 0.065 | AT4G30880 | LTPL29/LTP family protein precursor | LOC_Os01g58660 |
| gi|115483839 | 0.138 | - | LTPL25/LTP family protein precursor | LOC_Os11g02350 |
| gi|115453181 | 0.098 | AT4G30880 | LTPL36/LTP family protein precursor | LOC_Os03g25350 |
| gi|297604659 | 0.101 | AT3G08770 | LTPL17/LTP family protein precursor | LOC_Os05g40010 |
| gi|902058 | 0.141 | - | LTPL26/LTP family protein precursor | LOC_Os11g02350 |
| gi|115471201 | 0.087 | - | LTPL169/LTP family protein precursor | LOC_Os07g12080 |
| gi|115456101 | 0.103 | - | LTPL28/LTP family protein precursor | LOC_Os03g59380 |
| gi|115440749 | 0.250 | AT3G08770 | LTPL16/LTP family protein precursor | LOC_Os01g60740 |
| gi|125584660 | 0.124 | AT1G66850 | LTPL151/LTP family protein precurso | LOC_Os03g02050 |
| gi|115471187 | 0.126 | - | LTPL164/LTP family protein precurso | LOC_Os07g11650 |
| gi|115471167 | 0.126 | - | LTPL166/LTP family protein precursor | LOC_Os07g11310 |
| gi|6525009 | 0.075 | - | heavy metal-associated domain containing protein | LOC_Os02g32814 |
| **signal transducer** | | | | |
| gi|297604635 | 0.245 | AT5G01300 | phosphatidylethanolamine-binding protein | LOC_Os05g39250 |
| gi|115441871 | 0.349 | AT2G43710 | acyl-desaturase | LOC_Os01g69080 |
| **Stress and defense** | | | | |
| gi|77552437 | 0.213 | AT1G28330 | auxin-repressed protein | LOC_Os11g44810 |
| gi|218191114 | 0.412 | - | glyceraldehyde-3-phosphate dehydrogenase | LOC_Os02g38920 |
| gi|115452337 | 0.267 | AT1G07890 | OsAPx1 - Cytosolic Ascorbate Peroxidase encoding gene 1-8 | LOC_Os03g17690 |
| gi|115474285 | 0.447 | AT3G09640 | OsAPx2 - Cytosolic Ascorbate Peroxidase encoding gene 4,5,6,8, | LOC_Os07g49400 |
| gi|115434810 | 0.476 | AT5G20080 | NADH-cytochrome b5 reductase | LOC_Os01g07910 |
| gi|115484681 | 0.484 | AT1G77120 | dehydrogenase | LOC_Os11g10480 |
| gi|14018051 | 0.489 | AT1G17290 | aminotransferase, classes I and II, domain containing protein | LOC_Os10g25130 |
| gi|115463191 | 0.479 | AT3G10920 | superoxide dismutase | LOC_Os05g25850 |
| gi|115444275 | 0.490 | - | ribulose bisphosphate carboxylase small chain | LOC_Os02g05830 |
| gi|10716615 | 0.350 | AT3G23490 | cyanate hydratase | LOC_Os10g33270 |
| gi|5360657 | 0.280 | AT5G05730 | anthranilate synthase component I-1 | LOC_Os03g61120 |
| gi|297598143 | 0.377 | AT2G36460 | fructose-bisphospate aldolase isozyme | LOC_Os01g67860 |
| gi|115447403 | 0.377 | - | phenylalanine ammonia-lyase | LOC_Os02g41630 |
| gi|297598143 | 0.377 | AT2G36460 | fructose-bisphospate aldolase isozyme | LOC_Os01g67860 |
| gi|29367419 | 0.359 | - | peptide methionine sulfoxide reductase msrB | LOC_Os03g24600 |
| gi|115464757 | 0.361 | AT5G04590 | ferredoxin-nitrite reductase | LOC_Os05g42350 |
| gi|4835588 | 0.444 | AT5G22300 | nitrilase | LOC_Os02g42350 |
| gi|46360428 | 0.445 | AT2G39800 | amino acid kinase | LOC_Os05g38150 |
| gi|125542290 | 0.396 | AT3G03190 | glutathione S-transferase | LOC_Os03g04260 |
| gi|46805452 | 0.402 | AT5G09650 | soluble inorganic pyrophosphatase | LOC_Os02g52940 |
| gi|51854423 | 0.425 | AT3G23600 | endo-1,3;1,4-beta-D-glucanase precursor | LOC_Os05g33100 |
| gi|115470072 | 0.443 | AT4G35090 | catalase isozyme B | LOC_Os06g51150 |
| gi|222632369 | 0.353 | AT5G62530 | expressed protein | LOC_Os12g40440 |
| gi|10716615 | 0.350 | AT3G23490 | cyanate hydratase | LOC_Os10g33270 |
| gi|115467154 | 0.339 | AT1G35720 | annexin | LOC_Os06g11800 |
| gi|115486753 | 0.256 | - | glycosyl hydrolase | LOC_Os11g47520 |
| gi|115441829 | 0.306 | AT4G14030 | selenium-binding protein | LOC_Os01g68770 |
| gi|75225211 | 0.293 | AT2G05710 | aconitate hydratase protein | LOC_Os08g09200 |
| gi|115456227 | 0.209 | - | expressed protein | LOC_Os03g60509 |
| gi|115479691 | 0.283 | AT2G22420 | peroxidase precursor | LOC_Os09g29490 |
| gi|55233175 | 0.235 | AT3G61440 | Cysteine synthase | LOC_Os04g08350 |
| gi|115480571 | 0.481 | AT3G03250 | UTP--glucose-1-phosphate uridylyl transferase | LOC_Os09g38030 |
| gi|115476190 | 0.282 | AT1G23740 | dehydrogenase | LOC_Os08g29170 |
| gi|115456826 | 0.230 | AT4G08900 | arginase | LOC_Os04g01590 |
| gi|190689248 | 0.247 | - | alpha-glucan phosphorylast isozyme | LOC_Os03g55090 |
| gi|115474715 | 0.457 | AT1G18450 | actin | LOC_Os08g04280 |
| gi|115472897 | 0.465 | AT3G63190 | ribosome recycling factor | LOC_Os07g38300 |
| gi|218188405 | 0.447 | AT3G04880 | DNA-damage-repair/toleration protein DRT102 | LOC_Os01g36090 |
| gi|24899397 | 0.411 | - | stress responsive protein | LOC_Os03g21040 |
| gi|51090748 | 0.466 | AT5G20720 | chaperonin | LOC_Os06g09679 |
| gi|115466024 | 0.262 | AT1G31812 | acyl CoA binding protein | LOC_Os06g02490 |
| gi|115476908 | 0.297 | AT3G52300 | expressed protein | LOC_Os08g37320 |
| gi|125536940 | 0.383 | AT5G63310 | nucleoside diphosphate kinase | LOC_Os12g36194 |
| gi|194033257 | 0.489 | AT2G07698 | ATP synthase | LOC_Os09g08910 |
| gi|115470215 | 0.129 | - | disease resistance response protein-like protein | LOC_Os07g01620 |
| gi|115470967 | 0.159 | AT2G14170 | aldehyde dehydrogenase | LOC_Os07g09060 |
| gi|115476134 | 0.121 | - | pathogenesis-related Bet v I family protein | LOC_Os08g28670 |
| gi|115452513 | 0.150 | - | pathogenesis-related Bet v I family rotein | LOC_Os03g18850 |
| gi|115458852 | 0.332 | AT1G24020 | pathogenesis-related Bet v I family protein | LOC_Os04g39150 |
| gi|306415981 | 0.134 | - | BBTI4 - Bowman-Birk type bran trypsin inhibitor precursor | LOC_Os01g03340 |
| gi|115484185 | 0.111 | AT3G10020 | expressed protein | LOC_Os11g05170 |
| gi|9757686 | 0.063 | - | small hydrophilic plant seed protein | LOC_Os01g06630 |
| gi|115465529 | 0.210 | AT1G05510 | DUF1264 domain containing protein | LOC_Os05g49440 |
| gi|115462209 | 0.252 | AT3G17020 | universal stress protein domain containing protein | LOC_Os05g06500 |
| gi|115463347 | 0.101 | AT2G40170 | small hydrophilic plant seed protein | LOC_Os05g28210 |
| gi|115467846 | 0.182 | - | late embryogenesis abundant group 1 | LOC_Os06g21910 |
| gi|115460202 | 0.184 | - | late embryogenesis abundant group 1 | LOC_Os04g49980 |
| gi|115475924 | 0.270 | - | late embryogenesis abundant group 1 | LOC_Os08g23870 |
| gi|158513346 | 0.353 | AT2G36640 | late embryogenesis abundant protein 1 | LOC_Os03g20680 |
| gi|115465191 | 0.396 | AT1G52690 | late embryogenesis abundant group 3 | LOC_Os05g46480 |
| gi|115460462 | 0.455 | AT4G21020 | late embryogenesis abundant group 3 | LOC_Os04g52110 |
| gi|115439457 | 0.311 | - | late embryogenesis abundant group 3 | LOC_Os01g50910 |
| gi|125542488 | 0.258 | - | late embryogenesis abundant protein D-34 | LOC_Os03g06360 |
| gi|115489678 | 0.294 | - | late embryogenesis abundant protein D-34 | LOC_Os12g43140 |
| gi|115445331 | 0.299 | - | late embryogenesis abundant domain- containing protein | LOC_Os02g15250 |
| **Protein folding and modification** | | | | |
| gi|115443599 | 0.183 | AT5G60640 | OsPDIL1-4 | LOC_Os02g01010 |
| gi|213959111 | 0.431 | - | DnaK family protein | LOC_Os03g16920 |
| gi|115440955 | 0.298 | AT3G09440 | DnaK family protein | LOC_Os01g62290 |
| gi|125542923 | 0.457 | AT2G32120 | DnaK family protein | LOC_Os03g11910 |
| gi|222631026 | 0.380 | AT4G24280 | DnaK family protein | LOC_Os05g23740 |
| gi|115448791 | 0.321 | AT4G25200 | heat shock 22 kDa protein | LOC_Os02g52150 |
| gi|312983209 | 0.119 | AT1G53540 | hsp20/alpha crystallin family protein | LOC_Os01g04370 |
| gi|115452113 | 0.068 | AT1G53540 | hsp20/alpha crystallin family protein | LOC_Os03g15960 |
| gi|1763972 | 0.130 | AT1G53540 | hsp20/alpha crystallin family protein | LOC_Os01g04360 |
| gi|125543097 | 0.076 | AT4G27670 | hsp20/alpha crystallin family protein | LOC_Os03g14180 |
| gi|115434946 | 0.180 | AT5G12020 | hsp20/alpha crystallin family protein | LOC_Os01g08860 |
| gi|115456397 | 0.449 | AT1G09210 | calreticulin precursor | LOC_Os03g61670 |
| gi|115471369 | 0.356 | AT1G09210 | calreticulin precursor protein | LOC_Os07g14270 |
| gi|115461741 | 0.342 | AT1G75270 | glutathione S-transferase | LOC_Os05g02530 |
| gi|600769 | 0.143 | AT2G16600 | peptidyl-prolyl cis-trans isomerase | LOC_Os02g02890 |
| gi|125562526 | 0.469 | AT3G48330 | protein-L-isoaspartate O-methyltransferase | LOC_Os08g44280 |
| gi|115472625 | 0.485 | AT4G05180 | oxygen evolving enhancer protein 3 domain containing protein | LOC_Os07g36080 |
| gi|115461585 | 0.448 | AT5G13120 | S-Cyclophilin | LOC_Os05g01270 |
| gi|115452301 | 0.087 | AT3G55040 | IN2-1 protein | LOC_Os03g17470 |
| gi|222625756 | 0.450 | AT2G39810 | expressed protein | LOC_Os03g52700 |
| gi|115465962 | 0.469 | AT2G35320 | eyes absent homolog 4 | LOC_Os06g02028 |
| gi|218188287 | 0.433 | AT3G50820 | oxygen-evolving enhancer protein 1 | LOC_Os01g31690 |
| **cell redox homeostasis 6** | | | | |
| gi|115470941 | 0.383 | AT1G19730 | thioredoxin | LOC_Os07g08840 |
| gi|115438617 | 0.432 | - | oxidoreductase | LOC_Os01g43090 |
| gi|297604606 | 0.443 | - | oxidoreductase | LOC_Os05g38230 |
| gi|115464453 | 0.045 | AT5G01670 | oxidoreductase | LOC_Os05g39690 |
| gi|115462001 | 0.280 | AT1G54870 | oxidoreductase | LOC_Os05g04870 |
| gi|75232618 | 0.322 | AT4G36400 | FAD-linked oxidoreductase protein | LOC_Os07g08950 |
| **protein metabolic process** | | | | |
| gi|55168089 | 0.139 | - | OsSCP28 | LOC_Os05g18604 |
| gi|115465685 | 0.270 | AT1G15000 | OsSCP31 | LOC_Os05g50600 |
| gi|222615915 | 0.262 | AT5G09640 | OsSCP56 | LOC_Os11g24510 |
| gi|222619232 | 0.187 | AT5G19740 | peptidase | LOC_Os01g54010 |
| gi|115440161 | 0.474 | AT5G65760 | OsProCP1 | LOC_Os01g56150 |
| gi|222624970 | 0.266 | AT1G14980 | chaperonin | LOC_Os03g25050 |
| gi|75244738 | 0.450 | AT3G02875 | hydrolase | LOC_Os07g14590 |
| gi|115459478 | 0.464 | AT5G54140 | hydrolase | LOC_Os04g44110 |
| gi|222619126 | 0.312 | - | dehydrin family protein | LOC_Os01g50700 |
| gi|21397263 | 0.413 | AT2G17630 | aminotransferase | LOC_Os03g06200 |
| gi|115445217 | 0.456 | AT2G30970 | aminotransferase | LOC_Os02g14110 |
| gi|115434860 | 0.458 | AT1G80360 | aminotransferase | LOC_Os01g08270 |
| gi|218198356 | 0.386 | AT2G30970 | aminotransferase | LOC_Os06g35540 |
| gi|115451029 | 0.259 | AT4G39660 | aminotransferase | LOC_Os03g07570 |
| gi|115455323 | 0.269 | - | cysteine synthase | LOC_Os03g53650 |
| gi|125537478 | 0.302 | AT3G22460 | cysteine synthase | LOC_Os12g42980 |
| gi|115442595 | 0.447 | AT2G43750 | cysteine synthase | LOC_Os01g74650 |
| gi|222618453 | 0.328 | - | glutathione S-transferase | LOC_Os01g27210 |
| gi|115487944 | 0.381 | AT4G24830 | argininosuccinate synthase | LOC_Os12g13320 |
| gi|108862549 | 0.293 | - | serine hydroxymethyltransferase | LOC_Os12g22030 |
| gi|51535759 | 0.405 | AT5G38530 | tryptophan synthase beta chain 2 | LOC_Os06g42560 |
| gi|222636322 | 0.454 | AT1G13900 | Ser/Thr protein phosphatase family protein | LOC_Os07g02090 |
| gi|115442163 | 0.248 | AT3G10150 | Ser/Thr protein phosphatase family protein | LOC_Os01g71420 |
| **carbohydrate metabolic process** | | | | |
| gi|115473645 | 0.459 | AT5G49650 | FGGY family of carbohydrate kinases | LOC_Os07g44660 |
| gi|9049411 | 0.373 | AT1G68560 | glycosyl hydrolase, family 31 | LOC_Os01g03950 |
| gi|218199495 | 0.290 | AT3G23640 | glycosyl hydrolase, family 31 | LOC_Os07g23880 |
| gi|115469496 | 0.418 | AT5G11720 | glycosyl hydrolase, family 31 | LOC_Os06g46284 |
| gi|115459584 | 0.460 | AT5G10560 | glycosyl hydrolase family 3 protein | LOC_Os04g44840 |
| gi|218191593 | 0.420 | AT1G78060 | glycosyl hydrolase family 3 protein | LOC_Os02g51620 |
| gi|115473973 | 0.392 | AT5G57655 | xylose isomerase | LOC_Os07g47290 |
| gi|78099751 | 0.450 | AT2G36460 | fructose-bisphospate aldolase isozyme | LOC_Os05g33380 |
| gi|115482534 | 0.301 | AT1G04410 | lactate/malate dehydrogenase | LOC_Os10g33800 |
| gi|222619163 | 0.352 | AT1G69830 | alpha-amylase precursor | LOC_Os01g51754 |
| gi|115465145 | 0.445 | AT1G72990 | beta-galactosidase 8 precursor | LOC_Os05g46200 |
| gi|218184128 | 0.318 | AT3G26720 | lysosomal alpha-mannosidase precursor | LOC_Os10g05069 |
| gi|218192500 | 0.389 | AT2G29560 | enolase | LOC_Os03g15950 |
| gi|115480295 | 0.280 | AT5G24400 | 6-phosphogluconolactonase | LOC_Os09g35970 |
| gi|222641750 | 0.322 | AT4G09020 | glycogen operon protein glgX | LOC_Os09g29404 |
| gi|18087684 | 0.440 | - | periplasmic beta-glucosidase precursor | LOC_Os03g53860 |
| gi|115454931 | 0.327 | AT1G23190 | phosphoglucomutase | LOC_Os03g50480 |
| gi|115446991 | 0.376 | AT1G66970 | Glycerophosphoryl diester phosphodieste - rase family protein | LOC_Os02g37590 |
| gi|115443927 | 0.396 | AT4G13430 | 3-isopropylmalate dehydratase large subunit 2 | LOC_Os02g03260 |
| gi|50511452 | 0.299 | AT1G65590 | beta-hexosaminidase precursor | LOC_Os05g34320 |
| gi|218198910 | 0.176 | - | 1,4-alpha-glucan-branching enzyme | LOC_Os06g51084 |
| gi|222636967 | 0.189 | - | alpha-galactosidase precursor | LOC_Os07g26900 |
| gi|115485699 | 0.207 | AT3G26720 | lysosomal alpha-mannosidase precursor | LOC_Os11g32260 |
| gi|262345463 | 0.228 | AT2G39930 | Alpha amylase | LOC_Os08g40930 |
| gi|115480303 | 0.344 | AT4G10750 | 4-hydroxy-2-oxovalerate aldolase | LOC_Os09g36030 |
| gi|218196491 | 0.236 | AT1G08110 | glyoxalase family protein | LOC_Os05g22970 |
| gi|125602450 | 0.234 | AT1G11840 | glyoxalase family protein | LOC_Os08g09250 |
| gi|115469436 | 0.248 | AT1G79550 | phosphoglycerate kinase protein | LOC_Os06g45710 |
| gi|115442283 | 0.451 | AT3G54440 | beta-galactosidase | LOC_Os01g72340 |
| gi|18542895 | 0.485 | AT3G17940 | aldose 1-epimerase | LOC_Os10g06720 |
| gi|222628985 | 0.270 | - | aldose 1-epimerase | LOC_Os04g38530 |
| gi|115454033 | 0.289 | AT3G02230 | alpha-1,4-glucan-protein synthase | LOC_Os03g40270 |
| gi|115451981 | 0.496 | AT2G28470 | beta-galactosidase precursor | LOC_Os03g15020 |
| gi|62733435 | 0.128 | AT5G51820 | phosphoglucomutase | LOC_Os10g11140 |
| **Other metabolism related proteins** | | | | |
| gi|218187662 | 0.324 | AT3G21360 | expressed protein | LOC_Os01g09430 |
| gi|125564136 | 0.334 | - | caffeoyl-CoA O-methyltransferase | LOC_Os09g30360 |
| gi|222616745 | 0.338 | AT1G21400 | dehydrogenase E1 component domain containing protein | LOC_Os12g08260 |
| gi|115478180 | 0.407 | AT5G48230 | acetyl-CoA acetyltransferase | LOC_Os09g07830 |
| gi|115452831 | 0.459 | AT3G10850 | metallo-beta-lactamase family protein | LOC_Os03g21460 |
| gi|125554856 | 0.377 | AT1G23800 | aldehyde dehydrogenase | LOC_Os06g15990 |
| gi|115468758 | 0.352 | AT3G24360 | enoyl-CoA hydratase/isomerase family protein | LOC_Os06g39344 |
| gi|115470781 | 0.355 | AT1G55510 | transketolase | LOC_Os07g07470 |
| gi|115455493 | 0.369 | AT1G14810 | semialdehyde dehydrogenase, NAD binding domain containing protein | LOC_Os03g55280 |
| gi|115472365 | 0.369 | AT3G20390 | endoribonuclease | LOC_Os07g33240 |
| gi|115458950 | 0.372 | AT1G14120 | gibberellin 20 oxidase 2 | LOC_Os04g39980 |
| gi|125571984 | 0.373 | - | NADP-dependent malic enzyme | LOC_Os01g54030 |
| gi|297596332 | 0.442 | - | DJ-1 family protein | LOC_Os01g11880 |
| gi|115487674 | 0.447 | AT1G21400 | dehydrogenase E1 component domain containing protein | LOC_Os12g08260 |
| gi|115461408 | 0.448 | AT3G48990 | AMP-binding domain containing protein | LOC_Os04g58710 |
| gi|297724299 | 0.449 | AT1G29850 | expressed protein | LOC_Os05g47446 |
| gi|125561647 | 0.408 | AT1G14220 | ribonuclease T2 family domain containing protein | LOC_Os08g33710 |
| gi|41053142 | 0.415 | AT1G75330 | ornithine carbamoyltransferase | LOC_Os02g47590 |
| gi|56784348 | 0.420 | AT1G74260 | phosphoribosylformylglycinamidine synthase | LOC_Os01g66500 |
| gi|75232919 | 0.431 | AT1G03475 | coproporphyrinogen III oxidase | LOC_Os04g52130 |
| gi|115476618 | 0.433 | AT2G24270 | aldehyde dehydrogenase | LOC_Os08g34210 |
| gi|115445929 | 0.460 | AT3G14390 | pyridoxal-dependent decarboxylase protein | LOC_Os02g24354 |
| gi|115436366 | 0.467 | AT3G16950 | dihydrolipoyl dehydrogenase | LOC_Os01g23610 |
| gi|115454137 | 0.467 | - | gibberellin 20 oxidase 2 | LOC_Os03g42130 |
| gi|50252009 | 0.472 | AT2G38700 | GHMP kinases ATP-binding protein | LOC_Os02g01920 |
| gi|5091608 | 0.479 | AT1G27680 | glucose-1-phosphate adenylyltransferase large subunit | LOC_Os05g50380 |
| gi|115435496 | 0.481 | AT5G63890 | histidinol dehydrogenase | LOC_Os01g13190 |
| gi|115448217 | 0.481 | AT1G71920 | aminotransferase | LOC_Os02g47940 |
| gi|115446799 | 0.482 | - | gibberellin receptor GID1L2 | LOC_Os02g35940 |
| gi|115467064 | 0.486 | - | CXE carboxylesterase | LOC_Os06g11090 |
| gi|115445929 | 0.460 | AT3G14390 | pyridoxal-dependent decarboxylase protein | LOC_Os02g24354 |
| gi|51535181 | 0.489 | AT1G66430 | kinase, pfkB family | LOC_Os06g12600 |
| gi|222624587 | 0.493 | AT4G26900 | imidazole glycerol phosphate synthasehisHF | LOC_Os03g15120 |
| gi|4760553 | 0.246 | AT5G14780 | erythronate-4-phosphate dehydrogenase domain containing protein | LOC_Os06g29180 |
| gi|115465273 | 0.271 | AT1G72810 | threonine synthase | LOC_Os05g47640 |
| gi|115451535 | 0.254 | - | nucleotide pyrophosphatase/phosphodiesterase | LOC_Os03g11530 |
| gi|115477633 | 0.239 | AT5G51970 | dehydrogenase | LOC_Os08g43190 |
| gi|115487332 | 0.230 | AT3G10020 | expressed protein | LOC_Os12g05210 |
| gi|218196143 | 0.232 | AT1G48030 | dihydrolipoyl dehydrogenase | LOC_Os05g06750 |
| gi|115466514 | 0.220 | - | GDSL-like lipase/acylhydrolase | LOC_Os06g06260 |
| gi|115454135 | 0.223 | AT2G19940 | semialdehyde dehydrogenase | LOC_Os03g42110 |
| gi|115459338 | 0.228 | AT5G14590 | dehydrogenase | LOC_Os04g42920 |
| gi|115468250 | 0.210 | - | PROLM24 - Prolamin precursor | LOC_Os06g31070 |
| gi|115476854 | 0.294 | - | alpha-amylase precursor | LOC_Os08g36900 |
| gi|115448547 | 0.294 | AT3G17810 | dihydroorotate dihydrogenase protein | LOC_Os02g50350 |
| gi|115461843 | 0.313 | AT3G45300 | acyl-coenzyme A dehydrogenase | LOC_Os05g03480 |
| gi|115477815 | 0.129 | AT3G23940 | dihydroxy-acid dehydratase | LOC_Os08g44530 |
| gi|222636543 | 0.112 | AT3G63410 | methyltransferase domain containing protein | LOC_Os07g08200 |
| gi|115466558 | 0.293 | AT4G01130 | GDSL-like lipase/acylhydrolase | LOC_Os06g06520 |
| gi|125558975 | 0.204 | AT3G26430 | GDSL-like lipase/acylhydrolase | LOC_Os07g39750 |
| gi|115471169 | 0.026 | - | Alpha-Amylase Inhibitors (AAIs) and Seed Storage (SS) Protein subfamily,allergenic | LOC_Os07g11330 |
| gi|115471181 | 0.094 | - | RAL6 - Seed allergenic protein RA5/RA14/RA17 precursor | LOC_Os07g11510 |
| gi|297721507 | 0.049 | - | Plant invertase/pectin methylesterase inhibitor | LOC_Os02g46290 |
| gi|297603262 | 0.320 | - | invertase/pectin methylesterase inhibitor family protein | LOC_Os04g49730 |
| gi|115440541 | 0.284 | - | cysteine proteinase inhibitor precursor protein | LOC_Os01g58890 |
| gi|297719583 | 0.043 | - | BBTI5 - Bowman-Birk type bran trypsin inhibitor precursor | LOC_Os01g03360 |
| gi|125602063 | 0.064 | - | glutelin | LOC_Os08g03410 |
| gi|115464709 | 0.044 | - | Alpha-glubulin | LOC_Os05g41970 |
| gi|41469581 | 0.169 | AT3G22640 | cupin domain containing protein | LOC_Os03g46100 |
| gi|218193892 | 0.152 | - | cupin domain containing protein | LOC_Os03g57960 |
| gi|115461739 | 0.472 | AT1G07750 | cupin domain containing protein | LOC_Os05g02520 |
| gi|115439131 | 0.136 | AT1G60740 | peroxiredoxin | LOC_Os01g48420 |
| gi|115459340 | 0.167 | AT5G63030 | OsGrx_C2.2 - glutaredoxin subgroup I | LOC_Os04g42930 |
| gi|115456623 | 0.495 | AT4G02930 | elongation factor Tu | LOC_Os03g63410 |
| gi|125596358 | 0.191 | AT5G46290 | 3-oxoacyl-synthase | LOC_Os06g09630 |
| gi|115467636 | 0.191 | - | anthocyanidin 3-O-glucosyltransferase | LOC_Os06g18670 |
| gi|125526269 | 0.192 | AT2G32520 | dienelactone hydrolase family protein | LOC_Os01g34700 |
| gi|125548166 | 0.086 | AT1G47980 | desiccation-related protein PCC13-62 precursor | LOC_Os04g33150 |
| gi|20161527 | 0.349 | AT2G21820 | seed maturation protein PM41 | LOC_Os01g46600 |
| gi|53792717 | 0.351 | AT4G29260 | HAD superfamily phosphatase | LOC_Os06g36400 |
| gi|108707474 | 0.185 | AT1G07645 | glyoxalase family protein | LOC_Os03g16940 |
| **Unknown / others** | | | | |
| gi|297723967 | 0.163 | - | caltractin | LOC_Os05g25620 |
| gi|537403 | 0.305 | - | dehydrin | LOC_Os03g45280 |
| gi|115488528 | 0.496 | - | amidohydrolase | LOC_Os12g28270 |
| gi|108707969 | 0.376 | AT5G58250 | expressed protein | LOC_Os03g21370 |
| gi|297597194 | 0.403 | AT4G32460 | expressed protein | LOC_Os01g42520 |
| gi|115480445 | 0.408 | AT5G66090 | expressed protein | LOC_Os09g36990 |
| gi|125553370 | 0.306 | AT5G52960 | expressed protein | LOC_Os05g49410 |
| gi|115477944 | 0.242 | - | expressed protein | LOC_Os09g02180 |
| gi|115482392 | 0.330 | AT1G07040 | expressed protein | LOC_Os10g32680 |
| gi|47777377 | 0.250 | - | expressed protein | LOC_Os05g30400 |
| gi|45736144 | 0.136 | - | expressed protein | LOC_Os08g04710 |
| gi|45736136 | 0.116 | - | expressed protein | LOC_Os08g04640 |
| gi|115474363 | 0.110 | AT3G12960 | expressed protein | LOC_Os08g01370 |
| gi|47497805 | 0.201 | AT3G07720 | kelch repeat protein | LOC_Os09g07460 |
| gi|149391461 | 0.270 | - | abscisic stress-ripening | LOC_Os11g06720 |
| gi|115435938 | 0.179 | AT2G36640 | embryonic protein DC-8 | LOC_Os01g16920 |
| gi|115435220 | 0.164 | AT1G17100 | SOUL heme-binding protein | LOC_Os01g11230 |
| gi|108706671 | 0.399 | AT2G18540 | cupin domain containing protein | LOC_Os03g10110 |
| gi|218188994 | 0.386 | AT1G29680 | DUF1264 domain containing protein | LOC_Os01g52830 |
| gi|115439929 | 0.268 | AT4G18920 | DUF1264 domain containing protein | LOC_Os01g54520 |
| gi|52077016 | 0.308 | - | coiled-coil domain-containing protein 72 | LOC_Os02g06450 |
| gi|115457002 | 0.275 | - | CBS domain containing membrane protein | LOC_Os04g05010 |
| gi|115449183 | 0.240 | AT2G24940 | cytochrome b5-like Heme/Steroid binding domain containing protein | LOC_Os02g55060 |
| gi|85699976 | 0.069 | - | AMBP1-Antimicrobial peptide MBP-1 family protein precursor | LOC_Os11g37270 |
| gi|115473615 | 0.319 | AT4G01870 | WD40-like Beta Propeller Repeat family protein | LOC_Os07g44410 |
| gi|218191863 | 0.482 | - | transposon protein | LOC_Os02g58170 |
| gi|77557101 | 0.319 | AT3G15790 | methyl-CpG binding domain containing protein | LOC_Os12g42550 |
| gi|213959194 | 0.459 | - | HMG1/2,sequence-specific DNA binding transcription factor activity | LOC_Os04g47690 |

**Supplementary Table S11:** Proteins downregulated by > 5.0-fold in *OsSar1* transgenic rice.

| **Accession no.^a^** | **Fold chang^b^** | **Orthologou^c^** | **Protein description** | **Loc no.^d^** |
| --- | --- | --- | --- | --- |
| **Transport proteins** | | | | |
| gi|115435714 | 0.166 | - | sar1c | LOC_Os01g15010 |
| gi|55296316 | 0.065 | AT4G30880 | LTPL29/LTP family protein precursor | LOC_Os01g58660 |
| gi|115483839 | 0.138 | - | LTPL25/LTP family protein precursor | LOC_Os11g02350 |
| gi|115453181 | 0.098 | AT4G30880 | LTPL36/LTP family protein precursor | LOC_Os03g25350 |
| gi|297604659 | 0.101 | AT3G08770 | LTPL17/LTP family protein precursor | LOC_Os05g40010 |
| gi|902058 | 0.141 | - | LTPL26/LTP family protein precursor | LOC_Os11g02350 |
| gi|115471201 | 0.087 | - | LTPL169/LTP family protein precursor | LOC_Os07g12080 |
| gi|115456101 | 0.103 | - | LTPL28/LTP family protein precursor | LOC_Os03g59380 |
| gi|125584660 | 0.124 | AT1G66850 | LTPL151/LTP family protein precurso | LOC_Os03g02050 |
| gi|115471187 | 0.126 | - | LTPL164/LTP family protein precurso | LOC_Os07g11650 |
| gi|115471167 | 0.126 | - | LTPL166/LTP family protein precursor | LOC_Os07g11310 |
| gi|6525009 | 0.075 | - | heavy metal-associated domain containing protein | LOC_Os02g32814 |
| **Stress and defense** | | | | |
| gi|115484185 | 0.111 | AT3G10020 | expressed protein | LOC_Os11g05170 |
| gi|115470967 | 0.159 | AT2G14170 | aldehyde dehydrogenase | LOC_Os07g09060 |
| gi|115470215 | 0.129 | - | disease resistance response protein-like protein | LOC_Os07g01620 |
| gi|115476134 | 0.121 | - | pathogenesis-related Bet v I family protein | LOC_Os08g28670 |
| gi|115452513 | 0.150 | - | pathogenesis-related Bet v I family protein | LOC_Os03g18850 |
| gi|306415981 | 0.134 | - | BBTI4 - Bowman-Birk type bran trypsin inhibitor precursor | LOC_Os01g03340 |
| gi|9757686 | 0.063 | - | small hydrophilic plant seed protein | LOC_Os01g06630 |
| gi|115463347 | 0.101 | AT2G40170 | small hydrophilic plant seed protein | LOC_Os05g28210 |
| gi|115467846 | 0.182 | - | late embryogenesis abundant group 1 | LOC_Os06g21910 |
| gi|115460202 | 0.184 | - | late embryogenesis abundant group 1 | LOC_Os04g49980 |
| **Protein folding and modification** | | | | |
| gi|115443599 | 0.183 | AT5G60640 | OsPDIL1-4 | LOC_Os02g01010 |
| gi|115452301 | 0.087 | AT3G55040 | IN2-1 protein | LOC_Os03g17470 |
| gi|600769 | 0.143 | AT2G16600 | peptidyl-prolyl cis-trans isomerase | LOC_Os02g02890 |
| gi|312983209 | 0.119 | AT1G53540 | hsp20/alpha crystallin family protein | LOC_Os01g04370 |
| gi|115452113 | 0.068 | AT1G53540 | hsp20/alpha crystallin family protein | LOC_Os03g15960 |
| gi|1763972 | 0.130 | AT1G53540 | hsp20/alpha crystallin family protein | LOC_Os01g04360 |
| gi|125543097 | 0.076 | AT4G27670 | hsp20/alpha crystallin family protein | LOC_Os03g14180 |
| gi|115434946 | 0.180 | AT5G12020 | hsp20/alpha crystallin family protein | LOC_Os01g08860 |
| **Metabolism** | | | | |
| gi|115471169 | 0.026 | - | Alpha-Amylase Inhibitors (AAIs) and Seed Storage (SS) Protein subfamily | LOC_Os07g11330 |
| gi|115471181 | 0.094 | - | RAL6 - Seed allergenic protein RA5/RA14/RA17 precursor | LOC_Os07g11510 |
| gi|297721507 | 0.049 | - | Plant invertase/pectin methylesterase inhibitor | LOC_Os02g46290 |
| gi|297719583 | 0.043 | - | BBTI5 - Bowman-Birk type bran trypsin inhibitor precursor | LOC_Os01g03360 |
| gi|125602063 | 0.064 | - | glutelin | LOC_Os08g03410 |
| gi|115464709 | 0.044 | - | Alpha-glubulin | LOC_Os05g41970 |
| gi|41469581 | 0.169 | AT3G22640 | cupin domain containing protein | LOC_Os03g46100 |
| gi|218193892 | 0.152 | - | cupin domain containing protein | LOC_Os03g57960 |
| gi|115439131 | 0.136 | AT1G60740 | peroxiredoxin | LOC_Os01g48420 |
| gi|115464453 | 0.045 | AT5G01670 | oxidoreductase | LOC_Os05g39690 |
| gi|115459340 | 0.167 | AT5G63030 | OsGrx_C2.2 - glutaredoxin subgroup I | LOC_Os04g42930 |
| gi|125596358 | 0.191 | AT5G46290 | 3-oxoacyl-synthase | LOC_Os06g09630 |
| gi|115467636 | 0.191 | - | anthocyanidin 3-O-glucosyltransferase | LOC_Os06g18670 |
| gi|125526269 | 0.192 | AT2G32520 | dienelactone hydrolase family protein | LOC_Os01g34700 |
| gi|125548166 | 0.086 | AT1G47980 | desiccation-related protein PCC13-62 precursor | LOC_Os04g33150 |
| gi|55168089 | 0.139 | - | OsSCP28 | LOC_Os05g18604 |
| gi|222636543 | 0.112 | AT3G63410 | methyltransferase domain containing protein | LOC_Os07g08200 |
| gi|222619232 | 0.187 | AT5G19740 | peptidase | LOC_Os01g54010 |
| gi|218198910 | 0.176 | - | 1,4-alpha-glucan-branching enzyme | LOC_Os06g51084 |
| gi|222636967 | 0.189 | - | alpha-galactosidase precursor | LOC_Os07g26900 |
| gi|115477815 | 0.129 | AT3G23940 | dihydroxy-acid dehydratase | LOC_Os08g44530 |
| gi|62733435 | 0.128 | AT5G51820 | phosphoglucomutase | LOC_Os10g11140 |
| **Unknown / others** | | | | |
| gi|297723967 | 0.163 | - | caltractin | LOC_Os05g25620 |
| gi|45736144 | 0.136 | - | expressed protein | LOC_Os08g04710 |
| gi|45736136 | 0.116 | - | expressed protein | LOC_Os08g04640 |
| gi|115474363 | 0.110 | AT3G12960 | expressed protein | LOC_Os08g01370 |
| gi|115435938 | 0.179 | AT2G36640 | embryonic protein DC-8 | LOC_Os01g16920 |
| gi|115435220 | 0.164 | AT1G17100 | SOUL heme-binding protein | LOC_Os01g11230 |
| gi|85699976 | 0.069 | - | AMBP1 - Antimicrobial peptide MBP-1 family protein precursor | LOC_Os11g37270 |

^a^ Accession no. is the name of a gene in the NCBI (National Center for Biotechnology Information).

^b^ The values were calculated as the average ratio of 115, 117 (*OsSar1abc* RNAi) to 114, 116 (kitaake) label.

^c^ Orthologous is the name of homologue of Arabidopsis according to the RGAP (Rice Genome Annotation Project Database).

^d^ Loc no. is the locus name of a gene in the RGAP (Rice Genome Annotation Project Database).

**Supplementary Table S12.** Protein-upregulated preferential metabolic pathways were identified by KOBAS.

| **No.** | **KEGG pathway(s)^a^** | **KEGG ID** | **Background number^b^** | **Input number^c^** | ***p*-Value^d^** | **FDR-corrected**  ***p*-value** |
| --- | --- | --- | --- | --- | --- | --- |
| 1 | Proteasome | osa03050 | 64 | 11 | 8.3030e-8 | 0.000003 |
| 2 | Protein processing in endoplasmic reticulum | osa04141 | 201 | 16 | 0.000007 | 0.000167 |
| 3 | RNA transport | osa03013 | 146 | 7 | 0.020554 | 0.233842 |
| 4 | Aminoacyl-tRNA biosynthesis | osa00970 | 114 | 6 | 0.021192 | 0.233842 |
| 5 | Alpha-Linolenic acid metabolism | osa00592 | 33 | 3 | 0.026594 | 0.239349 |
| 6 | Phenylalanine, tyrosine and tryptophan biosynthesis | osa00400 | 41 | 3 | 0.044481 | 0.285952 |
| 7 | Fatty acid degradation | osa00071 | 41 | 3 | 0.044481 | 0.285952 |

^a^ All KEGG pathways were retrieved from KEGG (<http://www.genome.jp/kegg-bin/get_htext?query=03050&htext=br08901.keg&option=-a>).

^b^ No. of rice proteins located in various pathways.

^c^ No. of up-regulated proteins in various pathways.

^d^ Pathways with a *p*-value higher than 0.05 were not listed.
